# Clinical and immunological control of experimental autoimmune encephalomyelitis by tolerogenic dendritic cells loaded with MOG-encoding mRNA

**DOI:** 10.1186/s12974-019-1541-1

**Published:** 2019-08-15

**Authors:** Judith Derdelinckx, María José Mansilla, Maxime De Laere, Wai-Ping Lee, Juan Navarro-Barriuso, Inez Wens, Irene Nkansah, Jasmijn Daans, Hans De Reu, Aneta Jolanta Keliris, Johan Van Audekerke, Verdi Vanreusel, Zoë Pieters, Annemie Van der Linden, Marleen Verhoye, Geert Molenberghs, Niel Hens, Herman Goossens, Barbara Willekens, Patrick Cras, Peter Ponsaerts, Zwi N. Berneman, Eva María Martínez-Cáceres, Nathalie Cools

**Affiliations:** 1 Laboratory of Experimental Hematology, Faculty of Medicine and Health Sciences, Vaccine and Infectious Disease Institute (VaxInfectio), University of Antwerp, Antwerp University Hospital (UZA), Wilrijkstraat 10, 2650 Edegem, Belgium; 2 Division of Neurology, Antwerp University Hospital, Edegem, Belgium; 3 Division of Immunology, Germans Trias i Pujol University Hospital and Research Institute, Campus Can Ruti, Badalona, Spain; 4 Department of Cellular Biology, Physiology and Immunology, Universitat Autònoma de Barcelona, Bellaterra, Cerdanyola del Vallès, Spain; 5 Center for Cell Therapy and Regenerative Medicine, Antwerp University Hospital, Edegem, Belgium; 6 Bio-Imaging Lab, University of Antwerp, Antwerp, Belgium; 7 Center for Statistics, I-Biostat, Hasselt University, Diepenbeek, Belgium; 8 Centre for Health Economics Research and Modelling Infectious Diseases (CHERMID), Vaccine and Infectious Disease Institute (Vaxinfectio), University of Antwerp, Antwerp, Belgium; 9 L-BioStat, I-BioStat, KU Leuven, Leuven, Belgium; 10 Laboratory of Medical Microbiology, Faculty of Medicine and Health Sciences, Vaccine and Infectious Disease Institute (VaxInfectio), University of Antwerp, Antwerp, Belgium; 11 Born Bunge Institute, Translational Neurosciences, Faculty of Medicine and Health Sciences, University of Antwerp, Antwerp, Belgium

**Keywords:** Messenger RNA electroporation, Tolerogenic dendritic cells, Antigen-specific treatment, Experimental autoimmune encephalomyelitis, Multiple sclerosis, Tolerance induction

## Abstract

**Background:**

Although effective in reducing relapse rate and delaying progression, current therapies for multiple sclerosis (MS) do not completely halt disease progression. T cell autoimmunity to myelin antigens is considered one of the main mechanisms driving MS. It is characterized by autoreactivity to disease-initiating myelin antigen epitope(s), followed by a cascade of epitope spreading, which are both strongly patient-dependent. Targeting a variety of MS-associated antigens by myelin antigen-presenting tolerogenic dendritic cells (tolDC) is a promising treatment strategy to re-establish tolerance in MS. Electroporation with mRNA encoding myelin proteins is an innovative technique to load tolDC with the full spectrum of naturally processed myelin-derived epitopes.

**Methods:**

In this study, we generated murine tolDC presenting myelin oligodendrocyte glycoprotein (MOG) using mRNA electroporation and we assessed the efficacy of *MOG* mRNA-electroporated tolDC to dampen pathogenic T cell responses in experimental autoimmune encephalomyelitis (EAE). For this, MOG_35–55_-immunized C57BL/6 mice were injected intravenously at days 13, 17, and 21 post-disease induction with 1α,25-dihydroxyvitamin D_3_-treated tolDC electroporated with MOG-encoding mRNA. Mice were scored daily for signs of paralysis. At day 25, myelin reactivity was evaluated following restimulation of splenocytes with myelin-derived epitopes. Ex vivo magnetic resonance imaging (MRI) was performed to assess spinal cord inflammatory lesion load.

**Results:**

Treatment of MOG_35–55_-immunized C57BL/6 mice with *MOG* mRNA-electroporated or MOG_35–55_-pulsed tolDC led to a stabilization of the EAE clinical score from the first administration onwards, whereas it worsened in mice treated with non-antigen-loaded tolDC or with vehicle only. In addition, MOG_35–55_-specific pro-inflammatory pathogenic T cell responses and myelin antigen epitope spreading were inhibited in the peripheral immune system of tolDC-treated mice. Finally, magnetic resonance imaging analysis of hyperintense spots along the spinal cord was in line with the clinical score.

**Conclusions:**

Electroporation with mRNA is an efficient and versatile tool to generate myelin-presenting tolDC that are capable to stabilize the clinical score in EAE. These results pave the way for further research into mRNA-electroporated tolDC treatment as a patient-tailored therapy for MS.

**Electronic supplementary material:**

The online version of this article (10.1186/s12974-019-1541-1) contains supplementary material, which is available to authorized users.

## Background

Multiple sclerosis (MS) is a chronic demyelinating disorder of the central nervous system (CNS), clinically reflected by episodes of neurological dysfunction. Extensive research over the past decades has revealed a key role for the immune system, with a complex attribution of both the innate and adaptive arms of immunity [1]. This knowledge drove the development of a wide armamentarium of disease-modifying therapies for MS, which are able to reduce autoimmune inflammation-driven clinical and radiological disease activity with varying efficacy. However, their immunomodulatory mode of action does not specifically tackle the dysfunctional immune response toward myelin, which is presumed to steer MS pathogenesis [2]. Moreover, this broader immune modulation can induce off-target effects, leading to adverse events such as opportunistic infections, secondary autoimmunity, and malignancies [3]. Thus, the need for more targeted, disease antigen-specific treatment strategies remains.

In this context, the use of myelin-specific tolerogenic dendritic cells (tolDC) is a promising strategy to re-establish tolerance in an antigen-specific manner. Indeed, a beneficial effect of intravenous administration of bone marrow-derived, 1α,25-dihydroxyvitamin D_3_ (vitD_3_)-treated tolDC loaded with myelin oligodendrocyte glycoprotein (MOG)_40–55_ peptide was demonstrated in a mouse model of experimental autoimmune encephalomyelitis (EAE) [4, 5]. Interestingly, there was no clear beneficial effect on the clinical score of EAE mice when they were treated with tolDC that were not loaded with myelin-derived peptides [4–6]. Similar findings have been demonstrated in other animal models of autoimmune diseases, including collagen-induced arthritis [7, 8] and autoimmune thyroiditis [9]. Altogether, these findings suggest that antigen loading is indispensable for optimal functioning of tolDC-based therapies.

The choice of antigen for tolDC loading is challenging due to different factors. First, it requires prior knowledge of the disease-related antigens. In the setting of MS, there are indications that the adaptive autoimmune reaction is directed toward myelin proteins, including MOG, myelin basic protein (MBP), and proteolipid protein (PLP) [2]. However, although immune-dominant myelin epitopes derived from various myelin proteins have been described in MS patients [10], the specific pattern of myelin reactivity is diverse and strongly patient-dependent [11, 12]. Moreover, there is strong evidence that the myelin-specific T cell reactivity in MS patients is dynamic over time, a phenomenon called epitope spreading [13–15]. This is believed to play an important role in the progression of both EAE and MS [14–16]. However, in contrast to animal models of MS, in which consistent epitope spreading cascades could be detected [17], the nature of epitope spreading in MS patients seems to be more complex [13] and further complicates the choice of target antigens for tolDC loading. In addition, one needs to carefully distinguish between naturally processed and cryptic epitopes. Indeed, even though reactivity toward certain so-called cryptic epitopes can be demonstrated ex vivo, these epitopes are not naturally processed and are thus not directly involved in disease pathogenesis [18–22]. Therefore, it can be hypothesized that induction of tolerance toward cryptic epitopes may have no beneficial effect on disease course, as was demonstrated by Anderton et al. who failed to prevent MBP-directed EAE by inducing tolerance toward the cryptic epitope MBP_89–101_ [21]. Lastly, many synthetic peptides are restricted to specific human leukocyte antigen (HLA) subtypes [23, 24] and therefore cannot be used irrespective of the HLA status of the donor, which limits their use for antigen loading of tolDC.

An elegant strategy to tackle the above-mentioned obstacles is to load DC with antigens using mRNA electroporation. This non-viral, non-DNA-based gene delivery method introduces mRNA encoding full-length proteins intracellularly by application of a low-voltage electrical field. Therefore, electroporation facilitates the presentation of multiple naturally processed epitopes by all HLA molecules of a particular patient [25–28], rather than a limited number of specific HLA-restricted epitopes. This strategy will allow broadening the repertoire of responding lymphocytes without the need for prior selection of specific epitopes to be targeted.

In the present study, we addressed the feasibility to generate murine MOG-presenting tolDC by mRNA electroporation. Furthermore, we aimed to provide proof-of-concept for the use of myelin mRNA-electroporated tolDC as a potential treatment for MS by assessment of the efficacy of *MOG* mRNA-electroporated tolDC to dampen pathogenic T cell responses in an animal model of MS, i.e., EAE. Ultimately, the use of mRNA electroporation to load tolDC with myelin antigens may offer the potential to tackle the dynamic and complex multi-epitope-targeted loss of tolerance in MS.

## Materials and methods

### Ethics

The procedures described in this study were approved by the Ethics Committee for Animals of the University of Antwerp (study number 2014-93 and 2017-08) or by the Ethics Committee on Animal Experimentation of the Germans Trias i Pujol Research Institute and by the Generalitat de Catalunya (protocol number 9469). All experiments were performed in strict accordance with EU and governmental regulations.

### Cell culture

The myeloid leukemia cell line K562 (American Type Culture Collection, Rockville, MD, USA) was cultured in culture medium consisting of Iscove’s modified Dulbecco’s medium (IMDM) supplemented with 10% fetal bovine serum (FBS; Life Technologies).

Murine dendritic cells (DC) were generated from bone marrow precursor cells as described previously by Mansilla et al. [4, 5]. Briefly, bone marrow cells from C57BL/6 mice were cultured following 8 days in complete medium consisting of Roswell Park Memorial Institute (RPMI) 1640 medium (Life Technologies, Thermo Fisher Scientific, Merelbeke, Belgium) supplemented with 4 mM l-glutamine (Life Technologies), 10 μg/mL gentamicin (Life Technologies), 1 μg/mL amphotericin B (Life Technologies), 20% FBS (Life Technologies), 1 mM sodium pyruvate (Life Technologies), and 1000 IU/mL murine granulocyte macrophage colony-stimulating factor (GM-CSF) (Peprotech, London, UK) to generate DC. For the generation of tolDC, 1 nM vitamin D_3_ (vitD_3_, Calcijex, Abbott Laboratories, Turkey) was added to the culture medium. Half of the culture medium was replenished on days 2, 4, and 6 with complete medium. On day 7, cells were either left untreated for the generation of immature DC (iDC), or were stimulated for 24 h with 0.1 μg/mL lipopolysaccharide (LPS) (Invivogen, Toulouse, France) for the generation of mature DC (mDC) and tolDC. We refer to Additional file 1: Figure S1 for a graphical overview of the cell culture protocol.

### Plasmids and mRNA

The complementary DNA sequences of murine myelin oligodendrocyte glycoprotein (MOG) were modified for optimal codon use in murine cells and subcloned into a pST1-plasmid vector under the control of a T7 promotor and with the addition of a poly(A) tail (GeneArt, Thermo Fisher Scientific, Life Technologies, Merelbeke, Belgium). For lysosomal targeting of the translated protein, the *MOG* sequence was preceded by a lysosomal-targeting signal sequence (Sig) [29] and followed by the transmembrane and extracellular domain of the murine DC-lysosomal-associated membrane protein (LAMP)-3. Two plasmid vectors were generated: one encoding full-length murine MOG (amino acids 1-246) (Sig-MOG-LAMP) and one encoding extracellular murine MOG (amino acids 29–156) (Sig-extracellular MOG-LAMP) (see Additional file 2: Figure S2 for a graphical overview of the protein structure of MOG and Additional file 3: Figure S3 for the codon-optimized plasmid sequences). Additionally, DNA plasmids encoding enhanced green fluorescent protein (eGFP, pGEM4Z/EGFP/A64 vector [30] kindly provided by Dr. Eli Gilboa, Duke University Medical Center, Durham, NC, USA) or codon-optimized human C-C motif chemokine receptor 5 (CCR5) were used. All plasmids were propagated in *Escherichia coli* supercompetent cells (Stratagene, La Jolla, CA, USA) and plasmid DNA was purified using a NucleoBond® Xtra Midi EF kit (Macherey-Nagel, Düren, Germany). Next, plasmid DNA was linearized by *Sap*I digestion for the *MOG* and *CCR5* plasmids and by *Spe*I digestion for the *eGFP* plasmid. Subsequently, linearized plasmid DNA was used as a DNA template for in vitro transcription with a T7 in vitro transcription kit (mMessage mMachine T7 kit, Ambion, Life Technologies), according to the manufacturer’s protocol. All mRNA constructs were stored at − 20 °C at a concentration of 1 μg/μL.

### Antigen loading

On day 7 of the DC culture, cells were electroporated with mRNA encoding full-length or extracellular murine MOG, as described previously [31]. For this, cells were resuspended in Opti-MEM medium (Life Technologies) at a concentration of 5–50 × 10^6^ cells/mL. Electroporation was performed with a Gene Pulser Xcell™ Electroporation system (Bio-Rad, California, US) using a time constant pulse of 300 V for 7 ms and 1 μg mRNA per 10^6^ cells with a minimum of 5 μg of mRNA per electroporation. Non-electroporated tolDC and tolDC electroporated in the absence of mRNA (i.e., mock-electroporated) were used as control cells. For evaluation of the transfection efficacy following electroporation with *Sig*-*MOG*-*LAMP* and *Sig*-*extracellular MOG*-*LAMP* mRNA (mRNA length 1380 and 1023 nucleotides, respectively), K562 myeloid cells, mDC, and tolDC were electroporated with equal numbers of mRNA copies (i.e., 5 μg of *Sig*-*extracellular MOG*-*LAMP* mRNA and 6.75 μg *Sig*-*MOG*-*LAMP* mRNA per electroporation, representing 9.16 × 10^12^ RNA copies for both constructs). Following electroporation, cells were left untreated for the generation of iDC, or were stimulated with 0.1 μg/mL LPS for the generation of mDC and tolDC. Twenty-four hours later, transfected cells were used in further experiments or cryopreserved.

For the generation of MOG_35–55_ peptide-pulsed tolDC, cells were cultured for an additional 18 h in the presence of 10 μM murine MOG_35–55_ (MEVGWYRSPFSRVVHLYRNGK) (Immunostep, Salamanca, Spain) and subsequently cryopreserved.

### Cryopreservation conditions

DC or lymphocytes were cryopreserved in freezing medium containing FBS and 10% dimethyl sulfoxide (DMSO, Sigma-Aldrich, Bornem, Belgium). Cells were slowly frozen to − 80 °C at a cooling rate of − 1 °C/min by using a Mr. Frosty freezing container (Nalgene, Rochester, USA). After 24–72 h, cells were transferred to liquid nitrogen for long-term storage.

### qRT-PCR

Total RNA was extracted from the transfected cells 2 h following electroporation using an RNeasy Micro Kit (Qiagen, Düsseldorf, Germany), according to the manufacturer’s instructions. PCR was performed using SYBR Green technology (SsoAdvanced™ Universal SYBR® Green Supermix, Bio-Rad). Primer pairs specific for the codon-optimized MOG construct were used (forward 5′-TGAGAATCCAGAACGTGCG-3′, reverse 5′-TCCTCGACCTTCAGTTCCA-3′, Bio-Rad). Relative mRNA levels were normalized to levels of the reference gene glyceraldehyde-3-phosphate dehydrogenase (GAPDH). Experiments were performed using a CFX96 thermal cycler (Bio-Rad) and data analysis was performed using Bio-Rad CFX Manager version 3.1 and qbase+ (Biogazelle, Ghent, Belgium).

### Flow cytometry

Expression of MOG by the K562 cell line was assessed by flow cytometric analysis 2 h, 4 h, 24 h, 96 h following electroporation using intracellular staining with the anti-MOG Z2 primary antibody [32], kindly provided by prof. C. Linington (University of Glasgow, UK), and a rabbit anti-mouse Fab′-FITC secondary antibody (Dako, Agilent Technologies, Diegem, Belgium). Cells were analyzed on a BD FACScan flow cytometer (Becton Dickinson).

Immunophenotyping of DC was performed using the following fluorochrome-labeled anti-mouse monoclonal antibodies: anti-CD11c-PE-Cy7 (BD), anti-CD86-PE (eBioscience, Thermo Fisher Scientific), anti-CD40-APC (Biolegend, San Diego, United States), anti-MHC-II I/A I/E-Pacific Blue (Biolegend), anti-CD169-PE (Biolegend), and anti-F4/80-APC (eBioscience). Phenotypic analysis of splenocytes obtained from mice in the in vivo model was performed upon blocking with TruStain FcX™ (anti-mouse CD16/32) antibody (BioLegend) using the following fluorochrome-labeled anti-mouse monoclonal antibodies: anti-CD3-FITC (BioLegend), anti-CD4-V450 (BD), anti-CD25-PE (BioLegend), anti-CD19-FITC (BioLegend), anti-CD5-PE (BioLegend), anti-CD1d-Alexa Fluor 647 (BioLegend), anti-NK1.1-APC-Cy7 (BioLegend), anti-NKp46-PE (BioLegend), and anti-CD27-Alexa Fluor 647 (BioLegend). LIVE/DEAD™ Fixable Aqua Dead Cell Stain (Life Technologies, Thermo Fisher Scientific) was added to assess cell viability. Isotype-matched control monoclonal antibodies or Fluorescence Minus One (FMO) controls were used to determine non-specific background staining in the DC or the splenocytes phenotyping experiments, respectively. Cells were analyzed on a CyFlow® ML flow cytometer (Sysmex Partec, Norderstedt, Germany).

For analytical flow cytometry, at least 10^4^ events were measured. All results were analyzed using FlowJo software (Tree Star, Ashland, USA).

### EAE induction and in vivo administration of electroporated tolDC

Female C57BL/6JOlaHsd mice (8–10 weeks old) were purchased from Envigo (Horst, The Netherlands). Mice were housed in the animal house of the Germans Trias i Pujol Research Institute (Badalona, Spain) under standard light- and climate-controlled conditions, with standard chow and water provided ad libitum.

Mice were immunized subcutaneously with 100 μg MOG_35–55_ peptide (Immunostep) emulsified in Freund’s complete adjuvant (1:1) containing 4 mg/mL *Mycobacterium tuberculosis* (strain H37RA, Difco, Detroit, MI, USA). Mice received 250 ng pertussis toxin (Sigma Chemical, St. Louis, MO, USA) intravenously at days 0 and 2. All animals were examined daily for well-being and clinical signs by two blinded observers according to the following criteria: 0, asymptomatic; 0.5, loss of tone in the distal half of the tail; 1, loss of tone in the entire tail; 1.5, hind limb weakness; 2, hind limb paralysis; 2.5, hind limb paraplegia; 3, forelimb weakness; 4, quadriparesis; 4.5, severe quadriparesis; 5, quadriplegia; and 6, death. Endpoint criteria were established to minimize suffering and ensure animal well-being. To analyze the efficacy of the different treatment options, the number of responder mice was calculated, with a responder as previously defined by Mansilla et al. [4], i.e., a mouse showing < 1-point increase in the mean clinical score compared to their respective initial clinical score at first treatment administration (day 13 pi).

To predetermine the sample size for the in vivo experiment, an a priori ANOVA power analysis was performed using G*Power [33], following data from Mansilla et al. [5], with effect size = 0.51, α = 0.05, power = 85%, and number of groups = 4. This showed a required sample size of 13 mice per treatment group.

Mice were injected intravenously at the first day the mean clinical score exceeded 1.0, reflecting a clinically relevant disease onset in the majority of mice, (day 13 pi) with (i) 1 × 10^6^ *Sig*-*extracellular MOG*-*LAMP* mRNA-electroporated tolDC, (ii) 1 × 10^6^ MOG_35–55_-peptide-pulsed tolDC, (iii) 1 × 10^6^ non-antigen-loaded and non-electroporated tolDC, or (iv) PBS (vehicle control). Injections were repeated at day 17 and 21 pi. Mice were sacrificed at different time points during the experiment: (i) before EAE induction (*n* = 4), i.e., healthy mice; (ii) following EAE induction, but without treatment, at day 14 pi, i.e., immunized mice (*n* = 3); (iii) between the second and third vaccination, at day 20 pi, i.e., intermediate time point (*n* = 3 per treatment group); and (iv) at the end of the experiment, at day 25 pi, i.e., end point (*n* = 11 for the PBS-treated group and *n* = 10 for the other treatment groups) (Additional file 4: Figure S4). Following euthanasia, spleens and inguinal and axillary lymph nodes were obtained. Spinal cords were obtained from three mice per treatment group, representative of treatment group’s mean and range, and fixed in 4% paraformaldehyde (PFA).

### Magnetic resonance imaging

PFA-fixed spinal cords from three mice per treatment group were prepared for ex vivo magnetic resonance imaging (MRI) acquisition as described by Noristani et al. [34]. Briefly, spinal cords were soaked for 48 h in a 0.1 M PBS (10× PBS; pH 7.4) solution supplemented with a gadolinium-based MRI contrast agent (1% Dotarem, 0.5 mmol gadoteric acid/mL; Guerbet, France) in order to enhance the MRI contrast. Next for scanning, spinal cords were fixed in a 1 mL syringe using a gel containing 1.5% agar (Sigma-Aldrich, Belgium), 5% sucrose (Sigma-Aldrich, Belgium), and 1% Dotarem. MR images were acquired in the axial plane (approx. perpendicular on spinal cord) on a 7T MRI scanner (Bruker, Ettlingen, Germany), operating on the Paravision 6 software platform (Bruker, Ettlingen, Germany), using a quadrature transmit-only resonator 89/72 (Bruker, Ettlingen, Germany) combined with a receive-only four-channel array coil. For acquisition, a multi-slice single spin echo sequence was used with the following parameters: TR 1700 ms, TE 11 ms, 40 slices, TA 12 h, FOV 10 × 10 mm^2^, acquisition matrix 256 × 256, slice thickness 0.6 mm, no slice gap, number of slices 40, voxel resolution 40 × 40 × 600 μm^3^. Images were analyzed using MRIcron software [35]. Three blinded observers assessed the number of hyperintense white matter spots independently.

### MOG-specific proliferation assay

For evaluation of the MOG_35–55_-specific splenocyte reactivity, 2 × 10^5^ fresh splenocytes were restimulated with 5 μM MOG_35–55_ peptide (Immunostep) in 96-well plates for 3 days. Unstimulated splenocytes were used as a negative control. Splenocytes stimulated with 50 ng/mL phorbol myristate acetate (PMA) (Sigma-Aldrich) and 500 ng/mL ionomycin (Sigma-Aldrich) served as a positive control. After 48 h, 50 μL of cell culture supernatant was collected and stored at − 20 °C for later cytokine secretion analysis. Next, 1 μCi of [^3^H]-thymidin (PerkinElmer, Waltham, MA, USA) was added per well for an additional 18 h of culture. The stimulation index (SI) was calculated as the mean counts per minute (cpm) of antigen-stimulated conditions divided by the mean cpm of the non-stimulated conditions.

### Cytokine release assays

The secretion of interleukin (IL)-12p70 by DC was determined using a murine IL-12p70 (eBioscience) ELISA kit.

The concentration of IL-4, IL-17A, GM-CSF, IL-10, vascular endothelial growth factor (VEGF)-A, IL-13, and tumor necrosis factor (TNF)-α in the supernatant following restimulation of fresh splenocytes with MOG_35–55_ was quantified using a Meso Scale Discovery (MSD) mouse U-plex assay kit (Meso Scale Discovery, Rockville, MD, USA). The concentration of interferon (IFN)-γ was determined using an IFN-γ (Peprotech) ELISA kit. All assays were performed following the manufacturer’s instructions.

To assess the in vitro allogeneic T cell-stimulatory capacity of DC, splenocytes obtained from BALB/C mice were stimulated with DC at a 10:1 ratio. Unstimulated splenocytes served as negative control. As positive control, splenocytes were stimulated with 50 ng/mL PMA (Sigma-Aldrich) and 500 ng/mL ionomycin (Sigma-Aldrich). Following 5 days of coculture, the level of IFN-γ secretion in the supernatant was determined by means of a murine IFN-γ ELISA kit (Peprotech) following the manufacturer’s instructions.

To assess the in vitro MOG-specific T cell stimulatory capacity following mRNA electroporation, 2.5 × 10^5^ thawed MOG_35–55_ reactive splenocytes were stimulated with 2.5 × 10^4^ of different mDC or tolDC conditions. Co-cultures were performed overnight (18 h) at 37 °C in a 96-well PVDF plate (Millipore, Bedford, MA, USA) coated with anti-IFN-γ antibody. Stimulation of splenocytes was quantified by murine IFN-γ ELISPOT (Mabtech, NackaStrand, Sweden), according to manufacturer’s instructions. Frequencies of IFN-γ-secreting cells were calculated based on the number of spots counted using an automated AID ELISPOT Reader system (AID GmbH, Strassberg, Germany) and analyzed using AID ELISPOT software version 5.0.

Screening for epitope spreading was performed with IFN-γ ELISPOT, as described above, on thawed splenocytes. A total of 2 × 10^5^ cells were stimulated overnight with 5 μM of myelin peptide at 37 °C. The following murine myelin peptides were used: MBP_4–14_, MBP_84–97_, PLP_139–151_, PLP_178–191,_ MOG_92–106_ (all from Anaspec, CA, USA, purchased through Eurogentec, Seraing, Belgium), PLP_56–70_ (Pepscan, Lelystad, The Netherlands), and MOG_35–55_ (Sigma-Aldrich) (see Additional file 7: Table S1). Unstimulated splenocytes served as a negative control. The following responder criteria were used: per 10^6^ splenocytes, the mean antigen-specific spot counts (i.e., the spot count in the peptide-stimulated condition) must be greater than or equal to 15 spots per well and at least 1.5 times as high as the background reactivity (i.e., the spot count in the negative control).

### Histological analysis

PFA-fixed spinal cord tissue was embedded longitudinally in PELCO® cryo-embedding compound (Ted Pella, Redding, CA, USA) and 10-μm-thick cryosections were made in a Leica CM1950 cryotome (Leica, Nussloch, Germany). Staining was performed on spinal cord slides using a classical hematoxylin-eosin (H&E) and a Luxol Fast Blue staining. Images were taken with an inverted Nikon Ti wide-field microscope equipped with automated stage and perfect focus system.

### Statistical analysis

**Fig. 1:**
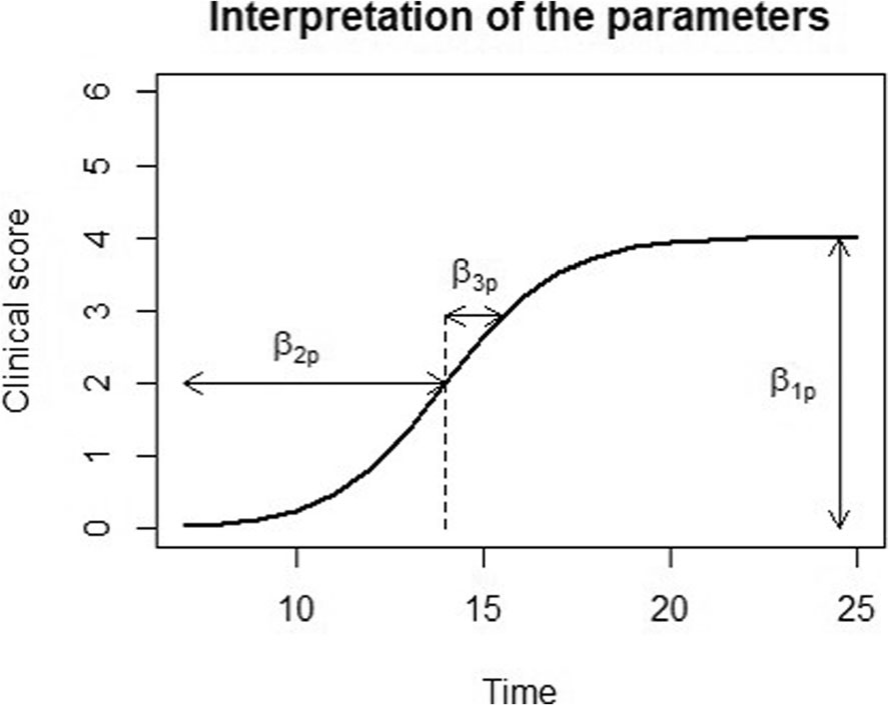
Interpretation of the model parameters. β_1p_ corresponds to the maximum clinical score, β_2p_ corresponds to the time at which half of the maximum clinical score is achieved, and β_3p_ corresponds to the steepness of the linear part of the curve. These features are allowed to vary depending on the treatment group *p* = 1, …,4 (Puls, Unpuls, mRNA, PBS)

Unless mentioned otherwise, results are shown as mean ± standard deviation (SD). Statistical analyses and graphical representations were performed with GraphPad Prism 5.0 software (GraphPad Software) and SAS 9.4. Statistical analysis was performed using parametric or non-parametric analyses, as appropriate, following Kolmogorov-Smirnoff analysis for normal distribution. To examine the difference in clinical score between the groups, a nonlinear model was designed, taking into account the longitudinal nature of the data and the missing data (introduced by sacrifice of mice during the course of the animal model) while providing a natural interpretation of the results. The model is described by the following equation:

$$ {y}_{ij}=\frac{\beta_{11} Puls+{\beta}_{12} Unpuls+{\beta}_{13} mRNA+{\beta}_{14} PBS+{u}_i}{1+\exp \left[-\frac{t_{ij}-\left({\beta}_{21} Puls+{\beta}_{22} Unpuls+{\beta}_{23} mRNA+{\beta}_{24} PBS\right)}{\beta_{31} Puls+{\beta}_{32} Unpuls+{\beta}_{33} mRNA+{\beta}_{34} PBS}\right]}+{\varepsilon}_{ij} $$

where *y*_*ij*_ is the clinical score for mouse *i* at time point *j* and *t*_*ij*_ corresponds to the time at which mouse *i* at time point *j* is scored. The error term *ε*_*ij*_ corresponds to the error in measurement for mouse *i* at time *j* and is independent and identically distributed (*i*.*i*.*d*.) *N*(0, *σ*^2^). The random effects *u*_*i*_ correspond to the additional change in clinical score for an individual mouse; hence, the random effects measure the deviation of each mouse from the average clinical score. *u*_*i*_ follows a $N\left(0,{\sigma}_u^2\right)$. Thus, the combination of Beta1p + *u*_*i*_ allow for a mouse-specific asymptotic clinical score. The error term *ε*_*ij*_ is independent and identically distributed (*i.i.d.*), *N*(0, *σ*^2^), and the random effects *u*_*i*_ follow a $N\left(0,{\sigma}_u^2\right)$. Figure 1 visualizes the interpretation of the model parameters. *β*_1*p*_ corresponds to the asymptotic clinical score, *β*_2*p*_ corresponds to the time at which half of the asymptotic clinical score is achieved, and *β*_3*p*_ corresponds to the steepness of the linear part of the curve. The subscript *p* allows that all the features can take on different values depending on the treatment group. Here, *p* = 1–4 since four treatment groups are compared (Puls, Unpuls, mRNA, PBS). At day 20, three mice from each group were sacrificed. The model is able to handle missing data under the assumption of missing at random (MAR), i.e., if, conditional on the observed data, missing values are not dependent on unobserved data [36]. Under the assumption of MAR, valid inference is obtained using a direct likelihood analysis, meaning the data is analyzed as is and no imputations are performed [36].

Of interest for the analysis is the parameter *β*_1*p*_. *β*_1*p*_ corresponds to the asymptotic clinical score; however, we will refer to *β*_1*p*_ as the maximum clinical score which in turn is related to disease severity. This approximation is justified since the function arrives closely to the asymptotic value in a short time interval. A difference in *β*_1*p*_ value between the treatment groups represents a difference in severity of disease. Holm’s procedure was used to correct *p*-values for multiple testing.

For all analyses, the *p* values were corrected using the appropriate procedure. The adjusted *p* value < 0.05 was considered as statistically significant.

## Results

### MOG mRNA electroporation of murine dendritic cells is an effective tool to induce long-term myelin expression and presentation

**Fig. 2:**
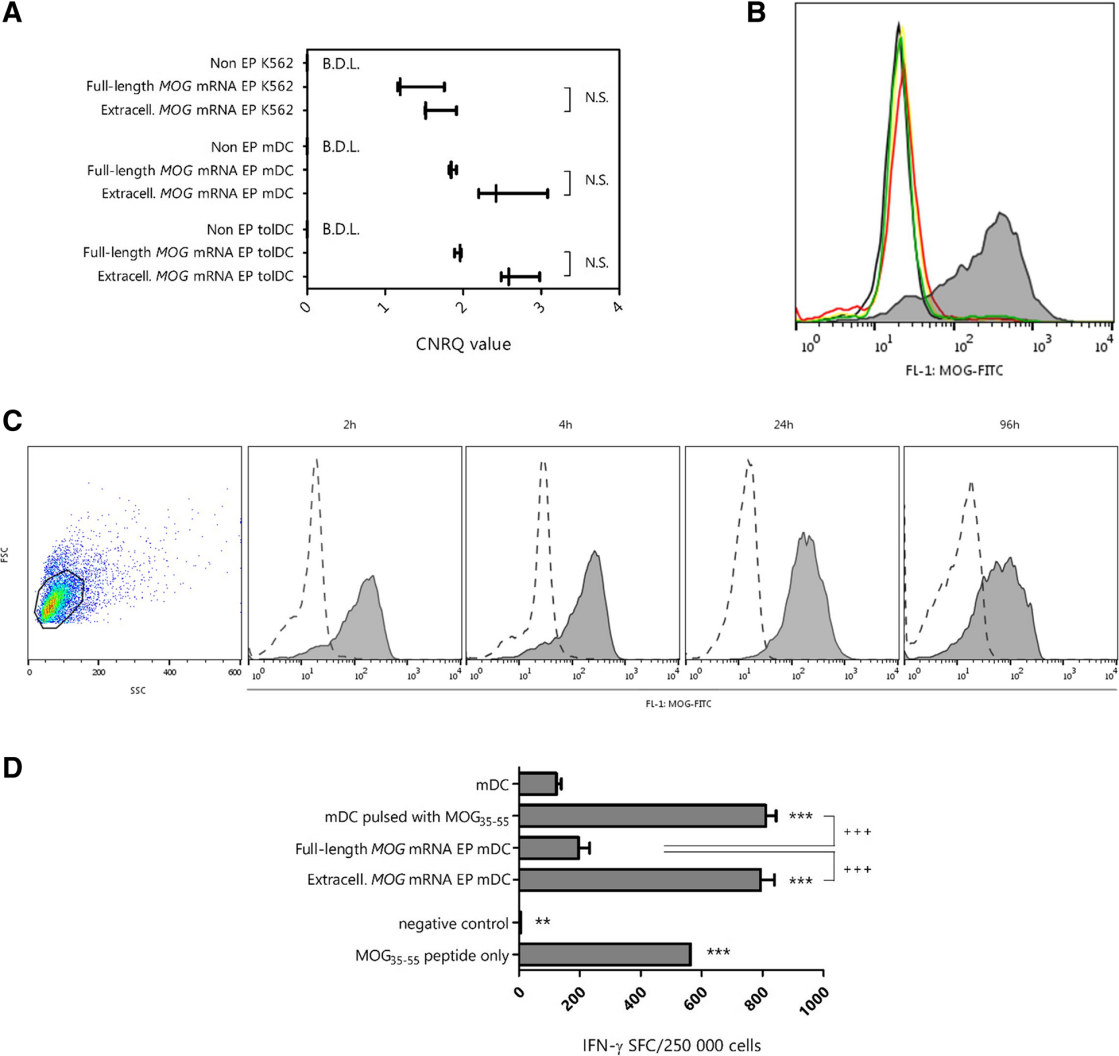
Induction of MOG expression and presentation by mRNA electroporation. **a** RT-qPCR analysis demonstrates intracellular presence of *Sig*-*MOG*-*LAMP* mRNA (Full-length *MOG*) and *Sig*-*extracellular MOG*-*LAMP* mRNA (Extracell. *MOG*), 2 h following electroporation. Results are shown as median log-transformed calibrated normalized relative quantity (CNRQ) values with minimum and maximum value (*n* = 3). Mann-Whitney *U* test was used for statistical analysis. **b** Flow cytometric analysis of MOG expression in K562 cells 24 h following electroporation with full-length *MOG* mRNA (red line), extracellular *MOG* mRNA (filled gray histogram), or irrelevant mRNA (*CCR5* mRNA, green line). Controls are non-electroporated and mock-electroporated K562 cells (black line and yellow line, respectively). Representative histograms from one experiment are shown (*n* = 3). **c** Kinetics of MOG expression after electroporation of K562 with extracellular *MOG* mRNA (filled histogram) as compared to non-electroporated K562 cells (dotted line). Flow cytometric results from one representative experiment are shown (*n* = 3). **d** MOG presentation following mRNA electroporation. MOG_35–55_-reactive splenocytes were co-cultured with different conditions of mDC in a 10:1 ratio. Results are shown as mean and SD and groups are compared via one-way ANOVA with Bonferroni post-hoc test; *, statistically significant compared to mDC; ^+^, statistically significant comparisons between groups denoted by solid line; **/^++^, *p* < 0.01; ***/^+++^, *p* < 0.001. *non*-*EP* non-electroporated, *MOG* myelin oligodendrocyte glycoprotein, *CNRQ* Calibrated Normalized Relative Quantity, *B*.*D*.*L*., below detection limit, *N*.*S*. not significant, *SFC* spot-forming cells

To evaluate the feasibility of electroporation with mRNA encoding full-length and extracellular MOG, *MOG* mRNA levels were analyzed 2 h following mRNA electroporation in K562 myeloid cells, mature dendritic cells (mDC), and tolDC. *MOG* mRNA was detected using RT-qPCR in cells electroporated with full-length *MOG* as well as in cells electroporated with extracellular *MOG*, while no mRNA was detected in non-electroporated cells (Fig. 2a). No difference in intracellular mRNA levels could be observed between cells that were electroporated with the full-length and extracellular *MOG*. As this confirmed that electroporation is a successful strategy to introduce mRNA into DC, we next investigated whether electroporated *MOG* mRNA was also translated into MOG protein using flow cytometric analysis. MOG protein could be readily detected in K562 cells electroporated with mRNA encoding extracellular MOG, while no expression of MOG protein could be detected in K562 cells electroporated with full-length *MOG* mRNA (Fig. 2b). Expression of MOG protein following electroporation with mRNA encoding extracellular MOG was detected as early as 2 h following transfection, with an median of 84.8% (7.5% interquartile range) MOG-expressing cells, and lasted up to 96 h following electroporation (Fig. 2c).

In order to determine whether mRNA-electroporated DC could efficiently process the protein and efficiently present MOG peptide fragments, *MOG* mRNA-electroporated DC were co-cultured with splenocytes from MOG_35–55_-induced EAE mice. Non-electroporated mDC pulsed with MOG_35–55_ peptide served as a control for MOG presentation. As shown by the number of interferon (IFN)-γ-producing T cells quantified by means of ELISPOT analysis, extracellular *MOG* mRNA-electroporated mDC are able to present MOG antigen to MOG-reactive splenocytes to the same extent as non-electroporated mDC pulsed with MOG_35–55_ peptide (Fig. 2d). In contrast, no difference in the magnitude of the IFN-γ response could be detected following stimulation with full-length *MOG* mRNA-electroporated mDC in comparison to splenocytes stimulated with non-electroporated mDC, demonstrating no evidence for MOG presentation by full-length *MOG* mRNA-electroporated mDC (Fig. 2d).

In summary, our findings demonstrate that electroporation with extracellular MOG-encoding mRNA is a valid method to induce MOG expression. Moreover, mDC electroporated with extracellular *MOG* mRNA are capable to effectively present antigenic MOG peptides to T cells.

### Following MOG mRNA electroporation, vitamin D_3_-treated tolDC retain their maturation-resistant phenotype

**Fig. 3:**
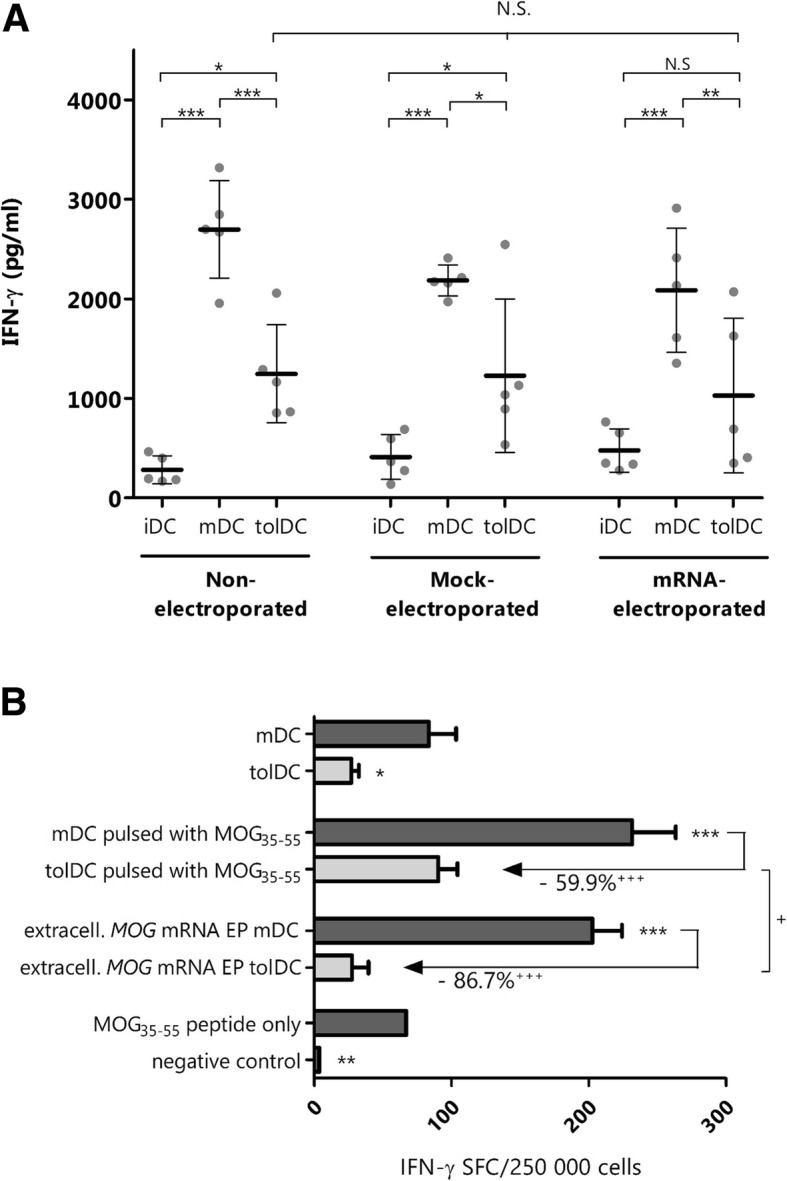
Modulatory capacity of *MOG* mRNA-electroporated tolDC. **a** IFN-γ secretion by allogeneic splenocytes following stimulation with DC (*n* = 5). Results are shown as mean ± standard deviation. *p < 0.05; **p < 0.01; ***p < 0.001 using two-way ANOVA with Bonferroni post-hoc test. Importantly, no differences in IFN-γ secretion could be detected between tolDC, mock-electroporated tolDC, and mRNA-electroporated tolDC mRNA. *N*.*S*. not significant. **b** Modulation of MOG-specific T cell responses by tolDC following electroporation with *MOG* mRNA. MOG_35–55_-reactive splenocytes were cocultured with different conditions of mDC and tolDC in a 10:1 ratio. Following overnight incubation, the number of IFN-γ-secreting T cells was quantified using ELISPOT. Results are shown as mean + standard deviation and groups are compared via a one-way ANOVA with Bonferroni post-hoc test. A sole asterisk (*) indicates the results is statistically significant compared to mDC; ^+^, statistically significant comparisons between groups denoted by solid line; **/^++^*p* < 0.01; ***/^+++^*p* < 0.001. *SFC* spot-forming cells, *N*.*S*. not significant, *extracell*. extracellular

As previously described [4, 5], treatment of murine DC with vitamin D_3_ (vitD_3_) results in the generation of tolDC, characterized by a maturation-resistant phenotype. Here, we demonstrate that treatment with vitD_3_ results in a significantly higher proportion of cells positive for the DC marker CD11c (Table 1). Moreover, upregulation of the expression of costimulatory markers CD40 and CD86 and of major histocompatibility complex (MHC) class II molecules following stimulation with pro-inflammatory lipopolysaccharide (LPS) was reduced in vitD_3_-treated tolDC as compared to mDC, reflected by a statistically significant difference in the fold change of the mean fluorescent intensity between mDC and tolDC as compared to immature DC (iDC) (Table 1). In addition, vitD_3_-treated tolDC secreted lower amounts of interleukin (IL)-12p70 following stimulation with pro-inflammatory LPS as compared to mDC (Table 1). Importantly, the reduced upregulation of the expression of costimulatory markers as well as the reduced secretion of IL-12p70 by tolDC following challenge with an pro-inflammatory product was not affected by mock or mRNA electroporation, except for the elevated expression of CD86 following mRNA electroporation of tolDC. Similarly, the capacity of tolDC to induce T cell hyporesponsiveness in an allogeneic mixed lymphocyte reaction was retained after mock and mRNA electroporation. Indeed, whereas mDC were strongly capable of inducing IFN-γ secretion by allogeneic splenocytes in co-culture, IFN-γ production was reduced when allogeneic splenocytes were stimulated with tolDC, irrespective of their electroporation status (Fig. 3a). Hence, the statistically significant increase of CD86 upregulation on tolDC following electroporation does not reflect a physiologically relevant phenomenon, as indicated by the absence of a significant correlation between CD86 MFI and IFN-γ secretion by tolDC (Spearman *r* = − 0.298, *p* = 0.347). Based on these findings, we conclude that mRNA electroporation does not affect the tolerogenic phenotype of vitD_3_-treated tolDC.

**Table 1:** Characterization of the tolerogenic phenotype of mRNA-electroporated tolDC

			iDC	mDC	tolDC	tolDC mock EP	tolDC mRNA EP
A. Immune phenotype							
Gated on viable cells	CD11c	% positive cells	45.2 (42.9–53.5)	42.1 (27.3–56.2)	73.5 (71.9–76.8)^*AA,BB*^	68.7 (66.0–80.1)^*A,B*^	68.0 (65.2–73.8)^*A*^
	CD169	% positive cells	12.0 (6.0–23.6)	22.5 (18.4–28.5)	57.5 (23.4–70.3)^*A*^	36.4 (20.5–65.7)	42.5 (19.6–69.5)
	F4/80	% positive cells	24.1 (16.4–31.4)	17.3 (11.8–27.6)	76.3 (30.7–82.2)^*B*^	70.4 (39.7–82.5)^*B*^	75.5 (39.7–82.5)^*A,B*^
Gated on viable CD11c^+^ cells	CD86	MFI	13.9 (9.0–24.9)^*B*^	55.1 (37.3–59.4)^*A*^	21.4 (16.8–26.5)	37.0 (29.2–52.0)	60.8 (46.6–71.2)^*AAA,CC*^
		Fold change in MFI compared to iDC	–	3.8 (1.7–4.8)	1.9 (0.8–2.6)^*D*^	2.3 (1.6–4.5)	3.3 (2.3–7.1)
	MHC-II	MFI	17.3 (9.3–28.8)	19.85 (10.6–27.0)	4.8 (2.1–5.6)^*A*,*B*^	4.7 (2.3–5.3)^*A*,*B*^	3.6 (2.5–5.6)^*A*,*B*^
		Fold change in MFI compared to iDC	–	1.2 (0.9–1.5)	0.3 (0.1–0.5)^*DD,BB*^	0.3 (0.1–0.4)^*B*^	0.3 (0.1–0.4)^*B*^
	CD40	MFI	2.5 (1.6–2.8)^*BBB*^	12.8 (11.0–21.3)^*AAA*^	7.5 (5.7–10.1)^*A*^	6.0 (4.7–9.4)	7.3 (5.2–9.1)^*A*^
		Fold change in MFI compared to iDC	–	6.0 (4.4–7.8)	2.8 (2.3–5.0)^*D,B*^	2.9 (2.2–3.6)^*BB*^	3.2 (2.8–3.9)^*B*^
B. Cytokine secretion profile							
IL-12p70 (pg/mL)			2.3 (1.5–5.8)^*BBB*^	356.3 (193.2–409.6)^*AAA*^	73.7 (61.9–132.9)^*BBB*^	74.3 (43.2–98.4)^*BBB*^	99.0 (48.1–122.3)^*BBB*^

A. Flow cytometric analysis of the expression of hallmark DC/macrophage and costimulatory markers by non-electroporated iDC, mDC, tolDC, and mock- or mRNA-electroporated tolDC (*n* = 8). mRNA-electroporated tolDC were electroporated with *eGFP* mRNA or full-length *MOG* mRNA. Results are shown as median (1st quartile-3rd quartile) of percentage of positive cells (compared to isotype control) or of mean fluorescent intensity (MFI). ^A^, statistically significant when compared to iDC; ^B^, statistically significant when compared to mDC; ^C^, statistically significant when compared to tolDC, all using Kruskal-Wallis test with Dunn’s post-hoc test; ^D^, statistically significant when compared to mDC using Mann-Whitney *U* test; ^A^/^B^/^C^/^D^*p* < 0.05; ^AA^/^BB^/^CC^/^DD^*p* < 0.01; ^AAA^/^BBB^/^CCC^/^DDD^*p* < 0.001. B. IL-12p70 secretion in the culture supernatant of iDC, mDC, and tolDC (*n* = 6). For this, DC were electroporated on day 6 of the cell culture protocol and stimulated on day 7 with a maturation cocktail consisting of 1 μg/mL LPS and 1000 IU/mL IFN-γ. Cell culture supernatant was harvested 24 h after addition of the maturation stimulus. Results are shown as mean ± standard deviation; ^A^, statistically significant when compared to iDC; ^B^, statistically significant when compared to mDC, using one-way ANOVA with Bonferonni’s post-hoc test; ^AAA^/^BBB^*p* < 0.001

### Extracellular MOG mRNA-electroporated tolDC effectively suppress MOG-specific splenocytes

In order to evaluate the capacity of extracellular *MOG* mRNA-electroporated tolDC to modulate MOG-specific T cell responses, splenocytes from MOG_35–55_ EAE mice were stimulated overnight with (i) non-antigen-loaded, non-electroporated mDC or tolDC, (ii) mDC or tolDC pulsed with MOG_35–55_, or (iii) mDC or tolDC electroporated with extracellular *MOG* mRNA. As depicted in Fig. 3b, extracellular *MOG* mRNA-electroporated tolDC displayed a reduced capacity to stimulate MOG-reactive splenocytes, as evidenced by a lower number of IFN-γ-producing cells in comparison with splenocytes stimulated with *MOG* mRNA-electroporated mDC. Similarly, MOG-specific splenocyte hyporesponsiveness was observed following stimulation with tolDC pulsed with MOG_35–55_, albeit, significantly less potently than with *MOG* mRNA-electroporated tolDC (Fig. 3b; 59.9 ± 12% vs 86.6 ± 5% reduction in IFN-γ spot-forming cells, respectively, *p* < 0.05). In conclusion, extracellular *MOG* mRNA-electroporated tolDC induce hyporesponsiveness of MOG-reactive splenocytes in vitro.

### In vivo administration of extracellular MOG mRNA-electroporated tolDC reduces EAE development

**Fig. 4:**
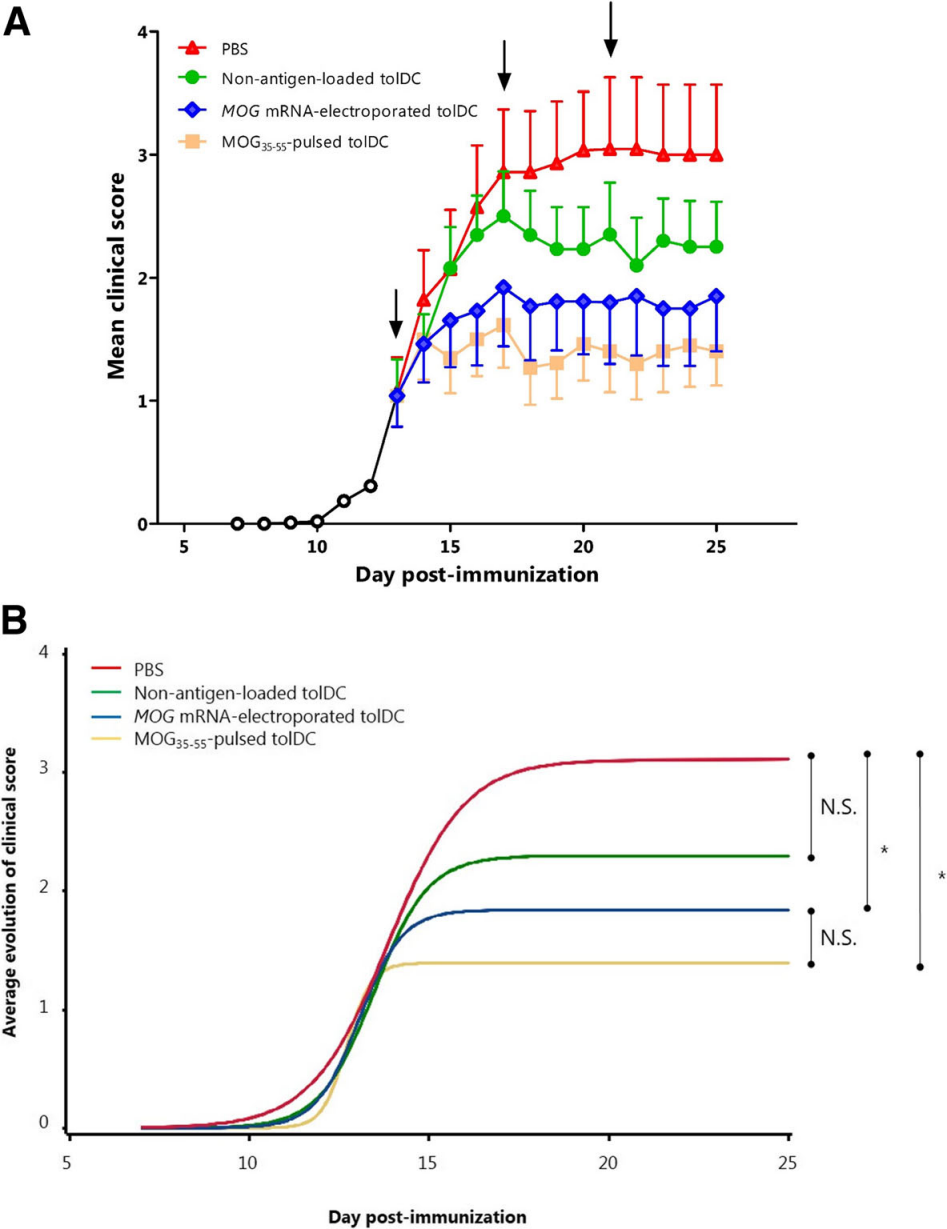
In vivo administration of extracellular *MOG* mRNA-electroporated tolDC abrogates EAE development. **a** Clinical follow-up of mice treated with 1 × 10^6^ viable non-antigen-loaded and non-electroporated tolDC (*n* = 13), MOG_35–55_-pulsed tolDC (*n* = 13), *MOG* mRNA-electroporated tolDC (*n* = 13) or PBS (*n* = 14). Arrows represent days of treatment (day 13 pi, day 17 pi, and day 21 pi). Results are represented as mean ± standard error of the mean. **b** Marginal average evolutions of the clinical score, as generated by the non-linear model described in the “Materials and methods” section; *p < 0.05. *N*.*S*. not significant, *PBS* phosphate-buffered saline

Next, we evaluated the therapeutic clinical effect of administering extracellular *MOG* mRNA-electroporated tolDC on EAE. For this, mice were injected intravenously as soon as the first clinical signs of disease appeared (day 13 post induction [pi]) with (i) 1 × 10^6^ extracellular *MOG* mRNA-electroporated tolDC, (ii) 1 × 10^6^ MOG_35–55_-pulsed tolDC, (iii) 1 × 10^6^ non-antigen-loaded, non-electroporated tolDC, or (iv) phosphate-buffered saline (PBS) (vehicle control). Mice were scored daily from day 7 pi onwards for signs of paralysis (Fig. 4a). Upon examining the severity in disease between the groups, a significant difference was observed for mice treated with *MOG* mRNA-electroporated (*p* = 0.04) and MOG_35–55_-pulsed tolDC (*p* = 0.01) compared to mice treated with PBS. No significant difference in severity of disease was observed between mice treated with non-antigen-loaded tolDC (*p* = 0.28) and PBS (Fig. 4b). No statistically significant difference in the clinical course between the *MOG* mRNA-electroporated and the MOG_35–55_-pulsed tolDC-treated groups could be observed (*p* = 0.53). We also determined the number of responder mice per group. As shown in Additional file 8: Table S2, a similar response rate (10/13, 76.9%) was obtained following treatment with both *MOG* mRNA-electroporated tolDC and MOG_35–55_-pulsed tolDC with more animals responding to the treatment as compared to the vehicle control group (4/14, 28.6%) or the non-antigen-loaded tolDC group (7/13, 53.8%). A graphical presentation of the responder mice per treatment group is depicted in Additional file 5: Figure S5.

In summary, these findings demonstrate that treatment with tolDC has a beneficial effect on the clinical course of EAE only when cells are loaded with antigen, either by using peptide-pulsing or mRNA electroporation. In both cases, the clinical score stabilized from the first administration onwards.

### TolDC treatment in EAE mice inhibits MOG-specific proliferation by splenocytes and lymph node-derived lymphocytes

**Fig. 5:**
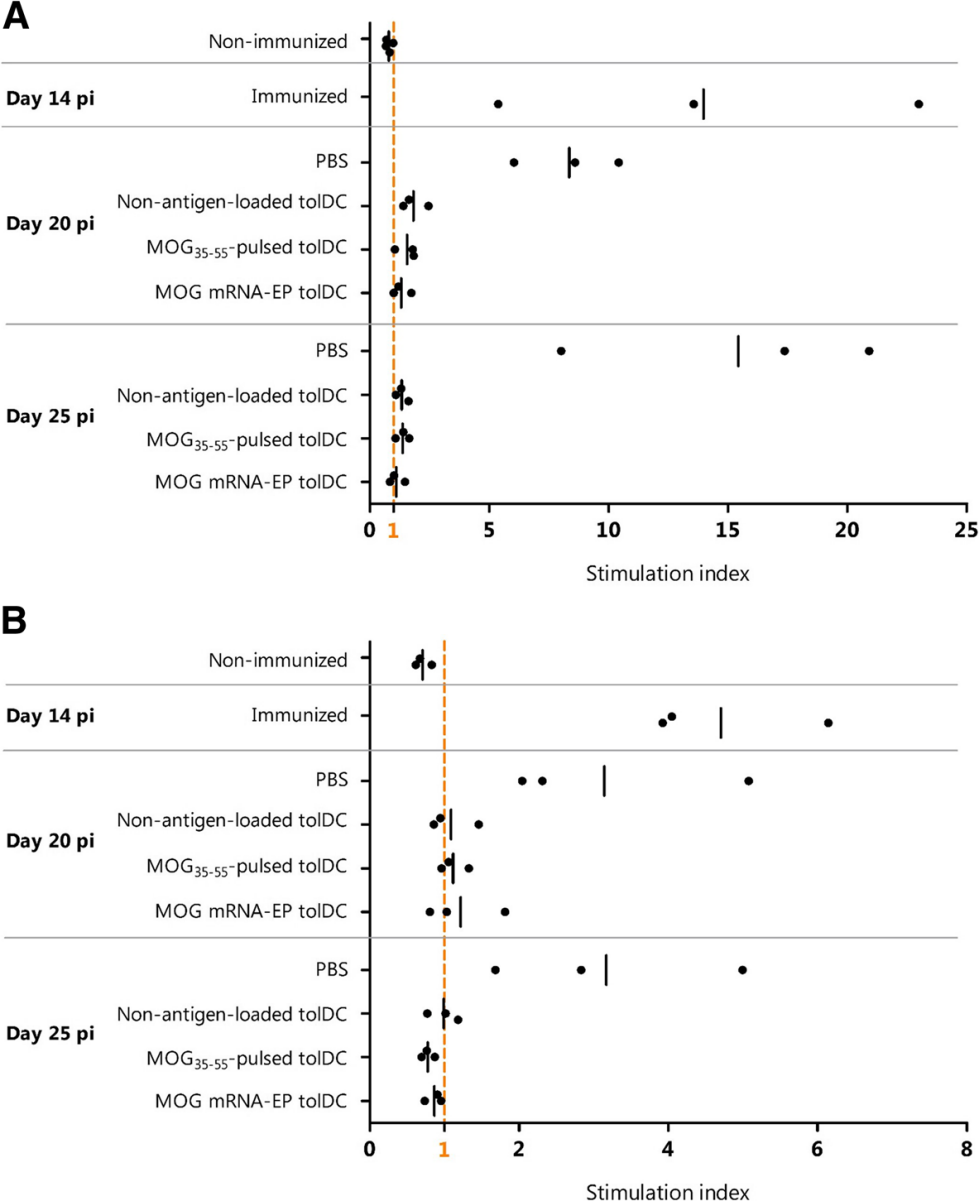
Myelin reactivity of splenocytes and lymph node cells obtained from tolDC- or PBS-treated EAE mice. MOG_35–55_-specific proliferation of splenocytes (**a**) and lymph node cells (**b**) at different time points during the in vivo EAE model. Cells were rechallenged with MOG_35–55_ or were left unstimulated as a negative control. The stimulation index was calculated as the mean counts per minute (cpm) of MOG_35–55_-stimulated conditions divided by the mean cpm of the non-stimulated conditions. The mean per treatment group is depicted as a vertical black line. *pi* post induction

MOG_35–55_-specific proliferation of fresh splenocytes and lymph node-derived lymphocytes was evaluated during the course of EAE. As shown in Fig. 5, no MOG_35–55_ antigen-specific proliferative reactivity could be demonstrated in healthy mice, whereas this response peaked during the first inflammatory phase of EAE (day 14 pi), following in vitro rechallenge of splenocytes (Fig. 5a) as well as of lymph node-derived lymphocytes (Fig. 5b) with MOG_35–55_. Treatment with tolDC inhibited MOG_35–55_-specific proliferation both at day 20 and day 25 pi.

### Epitope spreading can be detected in untreated and PBS-treated EAE mice, but not in tolDC-treated mice

Next, we evaluated the presence of intra- and intermolecular epitope spreading during the course of EAE. Cryopreserved splenocytes were restimulated with different myelin-derived peptides. The number of IFN-γ-secreting cells was evaluated by means of ELISPOT as a measure for antigen-specific stimulation (Table 2). Only a very low number of myelin-specific responder mice was observed among the healthy C57BL/6 mice, whereas immunized mice demonstrated a clear and focused MOG_35–55_-specific response at day 14 pi, corresponding with the rapid development of clinical symptoms. Interestingly, with further progression of the disease, intramolecular spreading to MOG_92–106_ occurs in PBS-treated mice at day 20 pi, but not in tolDC-treated mice, irrespective of antigen loading of the cells. Some PBS-treated mice also displayed intermolecular epitope spreading at this time point (one out of three mice for MBP_84–97_ and PLP_139–151_, two out of three mice for PLP_178–191_). Markedly, hardly any MOG_35–55_ reactivity or epitope spreading could be detected in tolDC-treated mice.

**Table 2:**
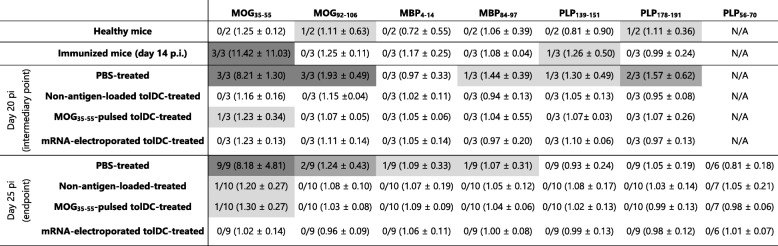
Myelin-specific IFN-γ ELISPOT responses at different time points in the EAE disease course

Number of responder mice is shown per myelin peptide, with between brackets the mean ratio of antigen-specific spot count over background spot count ± standard deviation. The following responder criteria were used: per 10^6^ splenocytes, the mean antigen-specific spot count must be greater than or equal to 15 spots per well and at least 1.5 times as high as the background reactivity (i.e., the spot count in the negative control). *pi* post induction

In conclusion, these data demonstrate that both intra- and intermolecular epitope spreading is present in the chronic MOG_35–55_ EAE model in a dynamic and time-dependent manner. Neither myelin reactivity nor epitope spreading could be detected in splenocytes of mice treated with *MOG* mRNA-electroporated tolDC, and only sporadically in mice treated with MOG_35–55_-pulsed tolDC or non-antigen-loaded tolDC.

### TolDC treatment effectively suppresses the strong EAE-mediated pro-inflammatory cytokine secretion

In order to assess the dynamics of the cytokine secretion profile in EAE, we determined the levels of eight different cytokines secreted by splenocytes collected at different time points during the disease and following rechallenge with MOG_35–55_ (Table 3). At the moment of EAE disease initiation, i.e., at day 14 pi, restimulation of splenocytes with MOG_35–55_ elicited strong T helper 17 (Th17) (IL-17A) and Th1 (IFN-γ, TNF-α) responses, known to be the major pro-inflammatory cytokines driving CNS inflammation in EAE. Additionally, a high secretion of the pro-inflammatory cytokine GM-CSF was detected, along with secretion of Th2 cytokines (IL-4 and IL-10; the latter known to have anti-inflammatory functions as well) to a lesser extent. The MOG_35–55_-induced pro-inflammatory cytokine secretion release reached its zenith at the end of the experiment in the PBS-treated mice. At this time point, secretion of VEGF-A and IL-13 could also be observed. Interestingly, treatment with tolDC strongly suppressed this MOG_35–55_-specific pro-inflammatory response. In conclusion, MOG_35–55_-directed splenocyte secretion profile in EAE is characterized by a strong Th17, Th1, and GM-CSF response, which is effectively suppressed by treatment with tolDC.

**Table 3:**
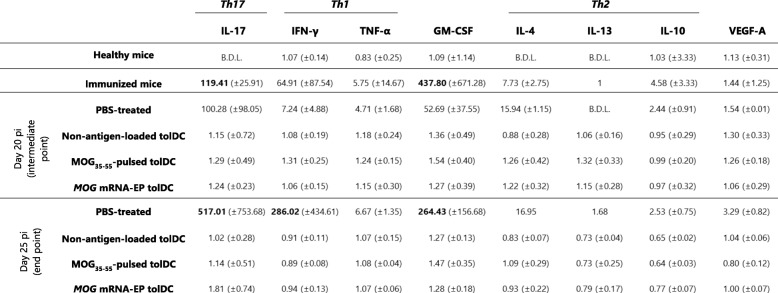
Cytokine secretion profile of splenocytes following restimulation with MOG_35–55_

The cytokine secretion profile upon restimulation with MOG_35–55_, with particular focus on Th1, Th2, and Th17 cytokines, was analyzed during the in vivo model. For this, mice from each treatment group were sacrificed at different time points, allowing to analyze the kinetics of cytokine secretion along the course of EAE. The fold increase in cytokine secretion following MOG_35–55_ restimulation was calculated by comparison to the negative control (unstimulated splenocytes). Results are expressed as the mean fold increase from three replicates ± standard deviation. In case the cytokine concentration was below the detection limit in the negative control, no fold increase could be calculated. Mean fold increases of > 2 log are indicated in bold. *EP* electroporation, *p*.*i*. post induction, *B*.*D*.*L*. protein concentration below detection limit in the MOG_35–55_-restimulated condition

### TolDC treatment attenuates spinal cord inflammatory lesion load

**Fig. 6:**
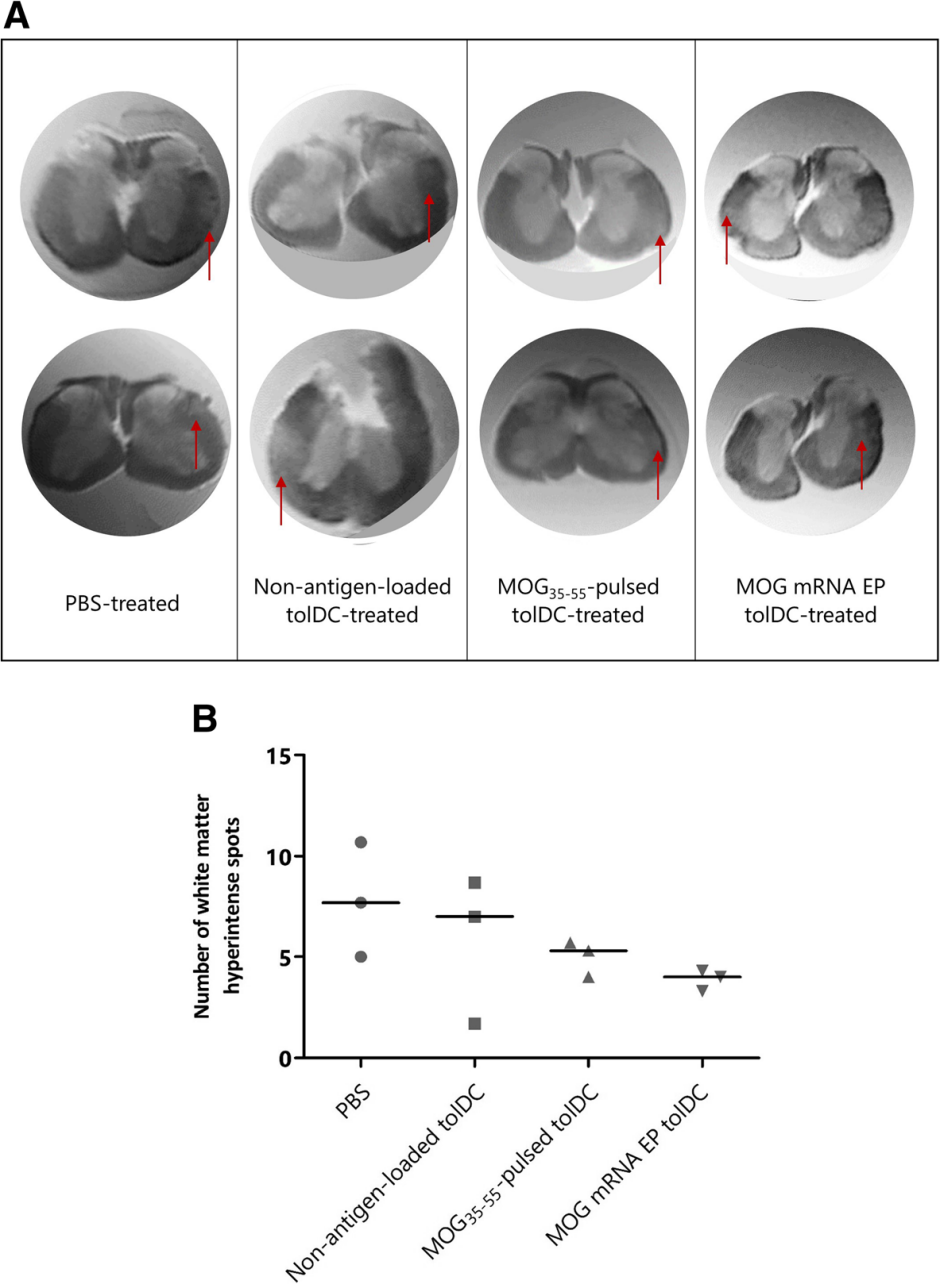
Evaluation of inflammatory lesion load within the spinal cord of tolDC-treated and PBS-treated mice using ex vivo MRI imaging. PFA-fixed spinal cords from three representative mice per treatment group were analyzed using MRI for the presence of white matter hyperintense lesions. **a** Representative MRI of spinal cord with hyperintense white matter spots marked with a red arrow. Two representative axial slices per treatment group are shown. **b** The total number of hyperintense white matter spots along the entire spinal cord was quantified as a measure of lesion load in three mice per treatment group. Results are presented as individual scores for hyperintense spots with median

**Fig. 7:**
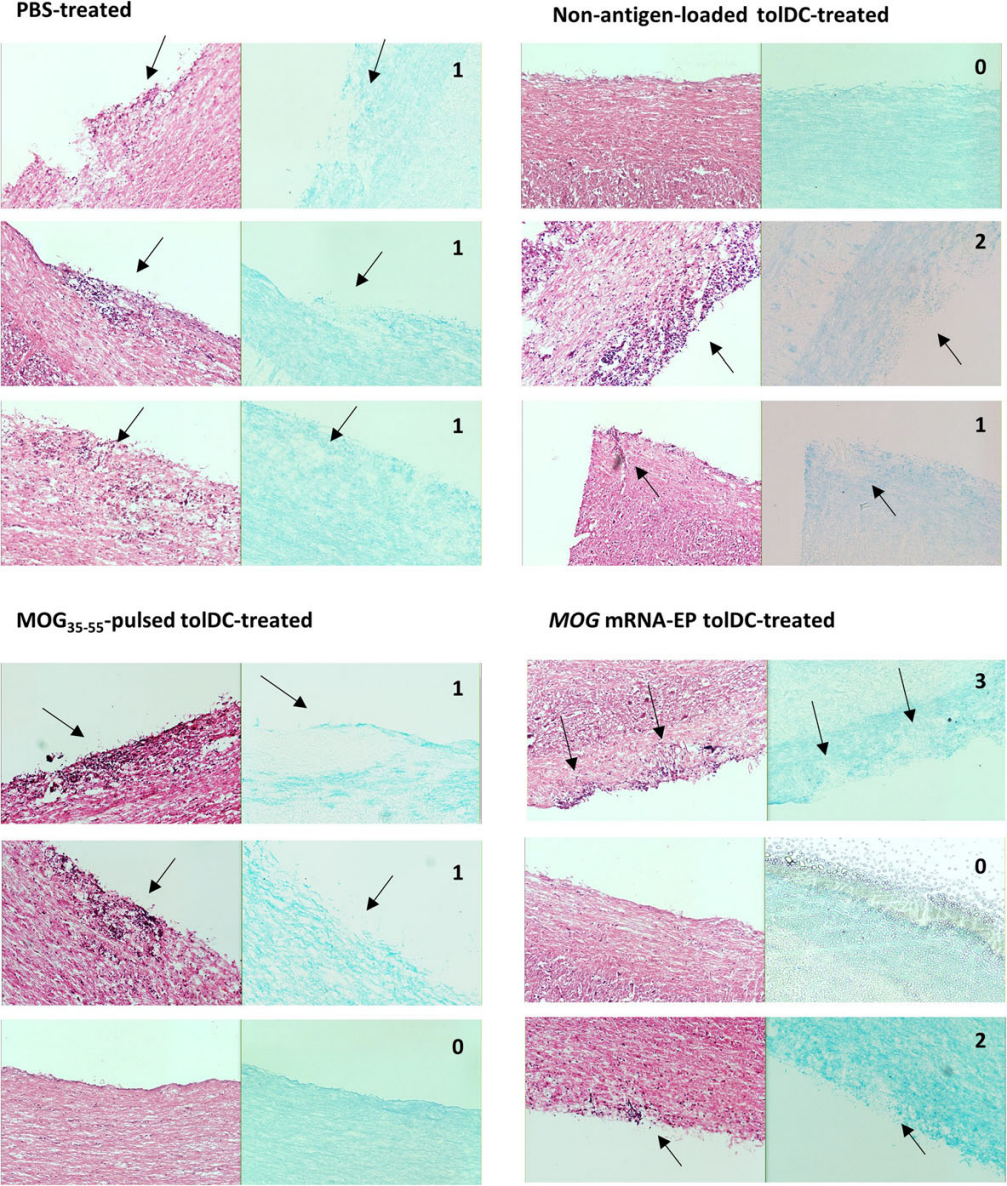
Hematoxylin-eosin (left panel) and Luxol Fast Blue (right panel) staining of spinal cord sections from mice either treated with PBS, with non-antigen-loaded tolDC, with MOG_35–55_-pulsed tolDC or with MOG mRNA-electroporated tolDC. For this, spinal cords were cryosected longitudinally at 20%, 40%, and 80% of depth. Arrows indicate the location of spinal cord demyelinization lesions. In the right upper corner of each panel group, the number of sections on which lesions could be identified is mentioned

Magnetic resonance imaging of the spinal cord was performed in order to evaluate the effect of tolDC treatment on inflammatory lesion load within the central nervous system (CNS). For this, MR images from three mice per treatment group were analyzed for the presence of white matter hyperintense areas reflecting inflammatory lesions. The median number of hyperintense spots was 4.0 following treatment of EAE mice with *MOG* mRNA-electroporated tolDC, 5.3 with MOG_35–55_-pulsed tolDC, 7.0 with non-antigen-loaded tolDC, and 7.7 with PBS (Fig. 6). Additionally, the presence of demyelinated lesions on cryosections from spinal cord regions with a high lesion load on MRI scan was confirmed using hematoxylin-eosin staining (for cell infiltration) and Luxol Fast Blue staining (for demyelination) (Fig. 7).

## Discussion

Although the exact cause of the breach in tolerance toward myelin in MS is not yet fully understood, a regulatory role for DC has been suggested [37]. Indeed, driven by the important function of DC in central and peripheral tolerance [38–42], it has been demonstrated that DC in MS patients are in a pro-inflammatory state and have an altered, migratory phenotype as compared to DC from healthy individuals [37, 43]. In doing so, they may disturb the balance between immunity and tolerance. These findings underline the important role of DC in the pathogenesis of MS and substantiate the choice for modulated DC as promising players for an antigen-specific cell-based approach for the treatment of MS.

Irrespective of the cell type used for therapeutic vaccination of autoimmune diseases, whether or not to target the cell-based approach toward disease-associated antigens remains to be elucidated. Previously, others demonstrated the need for auto-antigen loading of tolDC for the treatment of EAE [4, 5, 44], collagen-induced arthritis [7, 8], and autoimmune thyroiditis [9]. In this study, we evaluated whether mRNA electroporation is an efficient method to induce myelin expression and presentation by DC. We demonstrated that MOG protein expression could be detected up to 4 days following mRNA electroporation. Moreover, MOG mRNA-electroporated DC was able to stimulate MOG_35–55_-reactive splenocytes, indicating antigen-presenting capacity of MOG mRNA-electroporated DC. The use of mRNA electroporation as an antigen-loading strategy promises to overcome several key issues associated with the selection of target antigens, including the need for prior knowledge of culprit antigens and the dynamic nature of antigen reactivity in autoimmunity, i.e., epitope spreading. Whereas epitope spreading is well-described in relapsing EAE models, including PLP_139–151_-induced EAE [45], it is less investigated in chronic MOG_35–55_ EAE. In this study, we demonstrated the presence of a targeted MOG_35–55_ immune response associated with the first clinical signs of disease, followed by both intra- and intermolecular epitope spreading in PBS-treated EAE mice in later stages of disease. Similarly, Van Zwam et al. detected intermolecular epitope spreading toward PLP_56–70_ and myelin-associated glycoprotein (MAG)_97–112_ in splenocytes obtained from MOG_35–55_-induced C57BL/6 EAE mice [46]. Interestingly, in our hands, initiation of the epitope spreading cascade could be prevented by early treatment with tolDC. Indeed, administration of tolDC when a targeted MOG_35–55_ response was present abrogated epitope spreading, reduced MOG_35–55_-specific splenocyte proliferation, and inhibited the pro-inflammatory response toward MOG_35–55_. Strikingly, also non-antigen-loaded tolDC were able to induce these immunological alterations. However, in the absence of antigen, they failed to result in a clinical benefit. This discrepancy between clinical results and immunological findings, as demonstrated by us and others [4, 5, 8], might point toward other antigen-specific mechanisms of tolerance induction responsible for clinical benefit. For instance, it has been demonstrated that amelioration of EAE following administration of MOG-loaded tolDC was related to expansion of splenic regulatory T cells (Treg) [5, 6], in contrast to treatment with unpulsed tolDC [5]. In our hands, however, no differences in number of Treg, regulatory B cells (Breg), or natural killer cells (NK) in the spleen population between the different treatment groups could be detected (Additional file 6: Figure S6). Likewise, Stoop et al. demonstrated that the inhibition of arthritis severity and progression following treatment with type II collagen-pulsed tolDC was not accompanied by an expansion of splenic Foxp3-positive Tregs, but by an increase in the number of IL-10-producing T cells and a decrease in IL-17-producing T cells [8]. The variety of methods and protocols available to detect antigen-specific T cell responses has complicated the interpretation of the results obtained from immunomonitoring approaches. Despite the overwhelming use of various immune assays, reported results are often met with skepticism caused mainly by two reasons: (i) high variability of results from the same laboratories and/or among different laboratories, and (ii) the lack of prioritization to report standardization, validation, and training strategies as well as assay criteria by the laboratories conducting immune testing. Hence, to further elucidate the underlying mechanisms that mediate tolDC-induced tolerance in vivo by MOG antigen-loaded tolDC or to demonstrate proof-of-concept of administering antigen-loaded tolDC, harmonization strategies for the immune assay protocols should be aimed for allowing meta-analysis for future experiments [47, 48].

Following electroporation with *Sig*-*MOG*-*LAMP* mRNA and *Sig*-*extracellular MOG*-*LAMP* mRNA, expression of MOG protein could only be demonstrated with the latter mRNA construct and only in the K562 cell line, not in murine dendritic cells, even though successful transfection was demonstrated using RT-qPCR. Possibly, this is due to differences in tertiary structure of the translated protein in K562 cells compared to DC, since the Z2 anti-MOG antibody recognizes a conformational epitope [49] and therefore differences in protein folding could interfere with proper binding of the antibody. Additionally, the *Sig*-*LAMP* mRNA sequence, flanking the MOG sequence, guides the MOG protein into the lysosomal pathway, hereby stimulating MHC class II presentation of MOG antigenic peptides [29]. This lysosomal pathway is particularly active in DC following stimulation with a maturation stimulus [50]. Hence, we hypothesize that the MOG protein is rapidly processed and presented by DC, hampering the demonstration of MOG protein expression following *Sig*-*MOG*-*LAMP* mRNA electroporation in DC. In our opinion, the demonstration of MOG presentation in *Sig*-*extracellular MOG*-*LAMP* mRNA-electroporated mDC and tolDC indirectly implies the presence of MOG expression. This is in line with previous studies by other authors [51], who were equally unable to directly demonstrate MOG expression in DC following transfection with nucleic acids encoding MOG, even though MOG presentation was present.

Additionally, we explored the effect of the different tolDC treatments on CNS inflammation. For this, the inflammatory lesion load was evaluated using MRI in an explorative experiment with spinal cord tissues from three mice per treatment group. This technique previously allowed to visualize inflammation-related spinal cord edema by increase of the T2 signal in EAE [52] and spinal cord injury [34]. In this study, our preliminary data of the MRI analysis of spinal cord tissue revealed a trend to reduction in the number of hyperintense spots in the spinal cord white matter in a small subgroup of tolDC-treated mice. Moreover, we confirmed the presence of demyelinated lesions by histological analysis of spinal cord regions with a high lesion load on MRI scans. This is in line with previous research showing a strong correlation between MRI and histological lesions [34]. Similar findings were described by Duraes et al., who demonstrated that clinical improvement of EAE following transfer of MOG_35–55_-loaded plasmacytoid DC (pDC) was associated with a reduction of histological spinal cord inflammatory foci [53]. This protective effect was mediated by recruitment and modulation of endogenous pDC and by inhibition of encephalitogenic T cells within the spinal cord following transfer and CNS migration of MOG antigen-loaded pDC, but was completely abolished upon transfer of unloaded pDC [53]. Therefore, both the migratory capacity to the inflamed CNS and disease antigen presentation appear to be crucial for the clinical effect of tolerogenic DC-based therapies. Although we did not perform tracking experiments to study the migration of tolDC following administration, migration to the CNS can be expected based on findings from Mansilla et al., who detected a transitory signal from murine NIR815 dye-labeled, vitD_3_-treated MOG_40–55_-loaded tolDC in the brain 24 h following administration [5]. Overall, our preliminary observations together with the findings by others raise the hypothesis that tolDC treatment mediates suppression of CNS inflammation in EAE. Since the need for antigen loading of tolDC for clinical benefit on the course of EAE was not reflected by a clear peripheral immunological mechanism, these findings may therefore point toward an immunological effect of antigen-loaded tolDC within the CNS. Hence, for future experiments, we suggest to focus on immunomonitoring within the CNS, e.g., the modulation of autoreactive T cells in spinal cord lesions, to further elucidate the underlying mechanisms that mediate tolDC-induced tolerance in vivo by MOG antigen-loaded tolDC.

In this study, we developed and used a new nonlinear statistical approach to model the clinical course of EAE. This method has distinct advantages over currently used methods [54]. Firstly, and in contrast to the Kruskal-Wallis test that is classically used for the statistical analysis of EAE data, there is no need to reduce the daily EAE scores to one summary score, such as mean, median, or area under the curve, to address the research question. Our model is capable of addressing differences in the extent of disease among treatment groups without combining the daily EAE scores in one summarizing score. Consequently, no information on the disease profiles is lost. Secondly, the model properly accounts for incompleteness in the data under the assumption of missing at random (MAR), making deletion of mice from the analysis unnecessary. Lastly, the model has good predictive ability of the individual disease profiles and provides a natural interpretation of the results. Nevertheless, there are also some drawbacks of this model, i.e., (1) the assumption of normality and homoscedasticity underlying the model are not met; (2) the predicted scores might potentially be larger than 6, since the clinical score is treated here as a continuous variable; and (3) although the model performs well in predicting the average clinical score, it does not reflect small fluctuations in the clinical score. However and taken together, our new nonlinear statistical approach is more appropriate to analyze the dynamic profile of EAE than the Kruskal-Wallis test. For the sake of completeness, we did compare the medians between the different treatment groups of EAE mice in our study using the Kruskal-Wallis test and reached the same statistical conclusions as with our new nonlinear model (data not shown).

There were a few limitations to this study. First of all, most of the ex vivo experiments have been performed in a small subgroup of animals. Secondly, although animals were observed for a considerable period following disease induction, an even longer period of follow-up could provide more insight into the long-term effect of tolDC treatment on the course of EAE. Thirdly, whereas a superior clinical effect of MOG antigen-loaded tolDC over non-antigen-loaded tolDC was demonstrated in this study, MOG_35–55_-pulsed tolDC and *MOG* mRNA-electroporated tolDC were equally effective. Conceptually, the hypothesized advantages of mRNA electroporation as an antigen loading technique—i.e., the induction of presentation of naturally processed epitopes in a HLA-independent manner, with the potential to tackle epitope spreading—will likely be most evident in the multi-epitope setting of MS. Indeed, presentation of additional epitopes by *MOG* mRNA-electroporated tolDC at the first signs of disease in the EAE model might be redundant, given the fact that administration of the cell-based vaccine was performed when only a focused response toward the disease-inducing epitope could be observed. In the human setting, however, myelin reactivity is more complex, with both disease-initiating epitope(s) and subsequent epitope spreading cascade being strongly patient-dependent [13, 15]. Therefore, one can hypothesize that loading of tolDC with one single epitope, although sufficient in the experimentally focused murine setting, may be insufficient in the human setting. Consequently, mRNA electroporation will probably reach its full value as antigen-loading technique in the multi-epitope setting of MS. For future research, we suggest additional preclinical assessments to validate our findings using relapsing-remitting EAE models which have been demonstrated to be associated with extensive epitope spreading, e.g., the PLP_139–151_ C57BL/6 EAE model [45] and the MBP SJL/J EAE model [55]. In doing so, the direct effect on epitope spreading of treatment with tolDC, electroporated with single or multiple myelin mRNA constructs, could be evaluated. Confirmation of the clinical benefit of mRNA-electroporated tolDC in various murine models of EAE, together with additional in-depth evaluation of the immunological mechanisms underlying this effect, would further substantiate the value of mRNA-electroporated tolDC as a tolerance-inducing treatment approach.

## Conclusion

In conclusion, we validated the mRNA electroporation technique in vitro as an efficient tool to generate myelin-presenting tolDC and demonstrated in vivo a clinical benefit of *MOG* mRNA-electroporated tolDC treatment in EAE mice. This protective effect was accompanied by a decrease in the MOG_35–55_-specific pro-inflammatory response in the peripheral immune system and was likely driven by suppression of CNS inflammation. Additionally, we extensively characterized the cytokine response and epitope spreading cascade in the chronic MOG_35–55_-induced EAE model. This study paves the way for additional research into mRNA-electroporated tolDC treatment in the human setting of MS, with patient-specific multi-epitope disease induction and progression. This will allow fully evaluating and appreciating the value of mRNA electroporation as antigen-loading technique with the potential of covering all potential epitopes and tackling epitope spreading.

## Additional files

Additional file 1: **Figure S1.** Graphical overview of the culture protocol for murine bone marrow-derived dendritic cells. (PDF 271 kb)

Additional file 2: **Figure S2.** Graphical overview of the protein structure of murine MOG. (PDF 357 kb)

Additional file 3: **Figure S3.** Nucleotide sequence of the *Sig-full length MOG-LAMP*-3 and *Sig-extracellular MOG-LAMP*-3 constructs. (PDF 385 kb)

Additional file 4: **Figure S4.** Graphical overview of the experimental set-up of the in vivo experiment. (PDF 279 kb)

Additional file 5: **Figure S5.** Representation of individual clinical scores. (PDF 349 kb)

Additional file 6: **Figure S6.** Flow cytometric evaluation of the proportion of Treg, Breg and NK cells in splenocytes. (PDF 289 kb)

Additional file 7: **Table S1.** Amino acid sequences of the myelin peptides used for screening for inter- and intramolecular epitope spreading. (PDF 317 kb)

Additional file 8: **Table S2.** Clinical data of MOG_35–55_ EAE mice. (PDF 393 kb)

## Data Availability

The datasets used and/or analyzed during the current study are available from the corresponding author on reasonable request.

## Abbreviations

BDL: Below detection limit
CCR5: C-C motif chemokine receptor 5
CNRQ: Calibrated normalized relative quantity
CNS: Central nervous system
cpm: Counts per minute
DC: Dendritic cell
EAE: Experimental autoimmune encephalomyelitis
eGFP: Enhanced green fluorescent protein
EP: Electroporated
FBS: Fetal bovine serum
GAPDH: Glyceraldehyde-3-phosphate dehydrogenase
GM-CSF: Granulocyte-macrophage colony-stimulating factor
HLA: Human leukocyte antigen
iDC: Immature dendritic cell
IFN-γ: Interferon gamma
IL: Interleukin
IMDM: Iscove’s modified Dulbecco’s medium
LAMP: Lysosomal-associated membrane protein
LPS: Lipopolysaccharide
MAR: Missing at random
MBP: Myelin basic protein
mDC: Mature dendritic cell
MOG: Murine oligodendrocyte glycoprotein
MRI: Magnetic resonance imaging
MS: Multiple sclerosis
MSD: Meso Scale Discovery
NS: Not significant
PBS: Phosphate-buffered saline
PFA: Paraformaldehyde
pi: Post induction
PLP: Proteolipid protein
PMA: Phorbol myristate acetate
RPMI: Roswell Park Memorial Institute medium
SD: Standard deviation
SFC: Spot-forming cells
SI: Stimulation index
Sig: Lysosomal-targeting signal sequence
TNF: Tumor necrosis factor
tolDC: Tolerogenic dendritic cell
VEGF: Vascular endothelial growth factor A
VitD_3_: 1α,25-Dihydroxyvitamin D_3_
