# Supplementary material for: Clinical and immunological control of experimental autoimmune encephalomyelitis by tolerogenic dendritic cells loaded with MOG-encoding mRNA
Source: J Neuroinflammation. 2019 Aug 15;16:167. doi: 10.1186/s12974-019-1541-1 (PMC6696692; doi:10.1186/s12974-019-1541-1)
Supplement: Supplementary file 1 — Figure S1. Graphical overview of the culture protocol for murine bone marrow-derived dendritic cells. (PDF 271 kb) [file 12974_2019_1541_MOESM1_ESM.pdf]

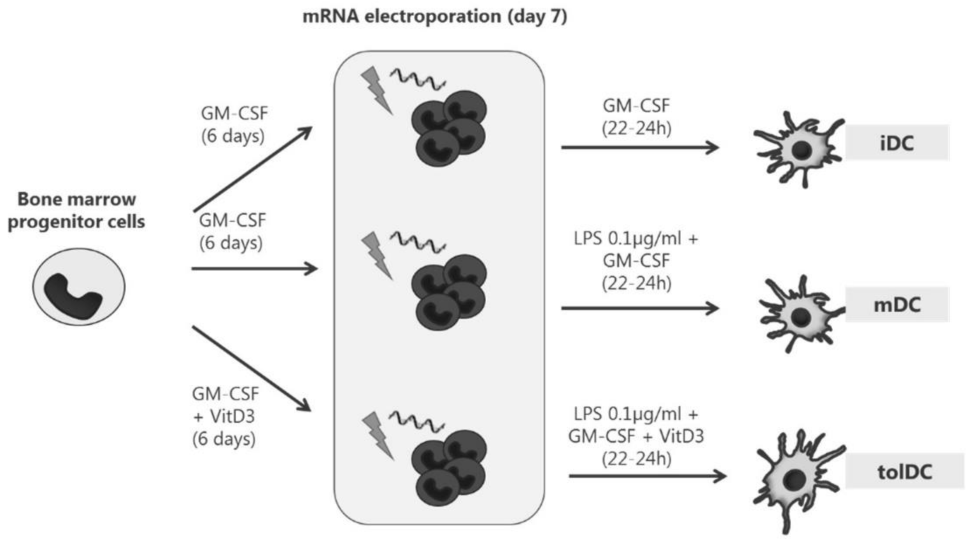

Supplementary Figure 1. Graphical overview of the culture protocol for murine bone marrow-derived dendritic cells.
