# Supplementary material for: Clinical and immunological control of experimental autoimmune encephalomyelitis by tolerogenic dendritic cells loaded with MOG-encoding mRNA
Source: J Neuroinflammation. 2019 Aug 15;16:167. doi: 10.1186/s12974-019-1541-1 (PMC6696692; doi:10.1186/s12974-019-1541-1)
Supplement: Supplementary file 2 — Figure S2. Graphical overview of the protein structure of murine MOG. (PDF 357 kb) [file 12974_2019_1541_MOESM2_ESM.pdf]

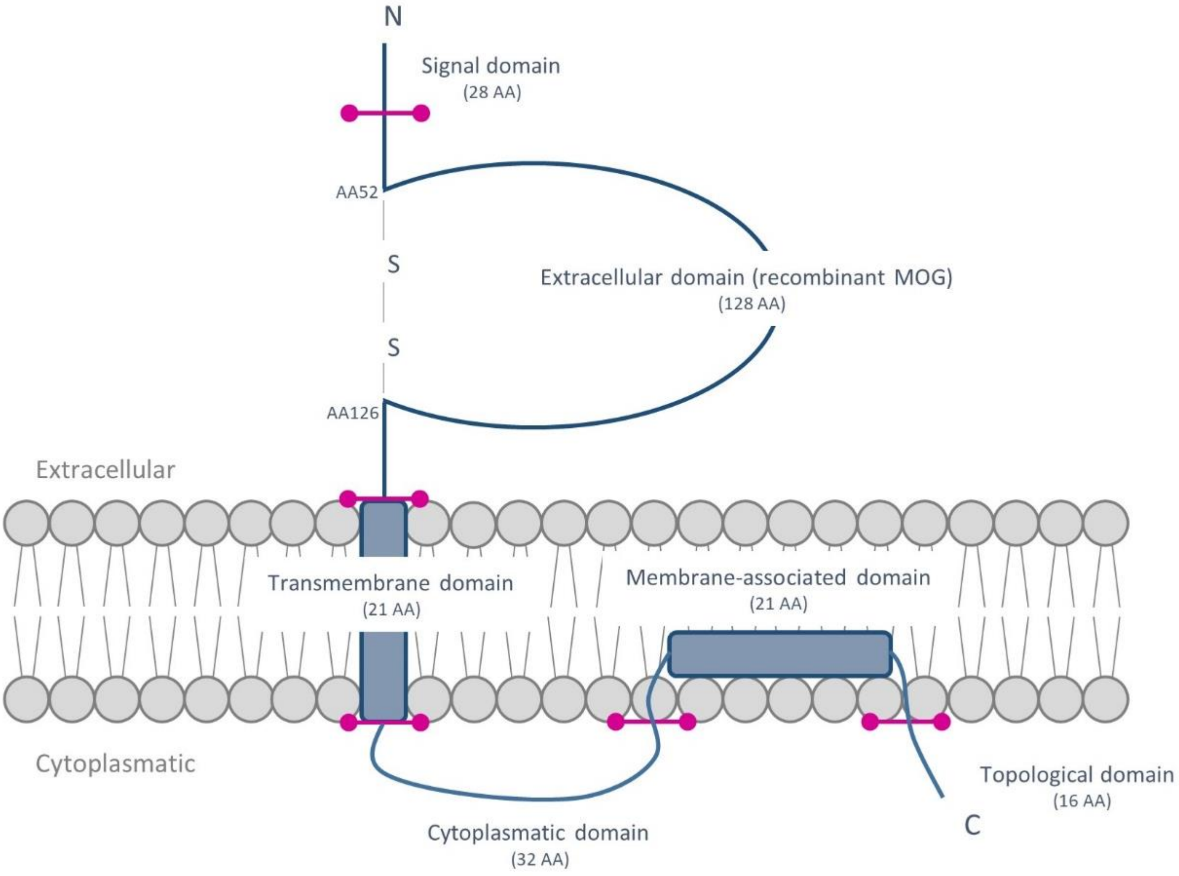

Supplementary Figure 2. Graphical overview of the protein structure of murine MOG, based on Kroepfl *et al.* (1) and Johns *et al.* (2). Although constituting only a minor part of the myelin sheath, MOG is a highly encephalitogenic protein capable of inducing EAE in rodents and primates (3). Moreover, MOG is a target antigen in human disease, as evidenced by the presence of MOG-directed T cell responses in MS patients (4, 5), as well as MOG antibodies in human demyelinating CNS diseases (6) and MS (5). This member of the Ig superfamily contains two hydrophobic membrane-associated domains, resulting in a large extracellular domain consisting of a signal sequence of 28 amino acids (AA), followed by a 128 AA extracellular domain. Purple lines represent boundaries between different domains. The preferential expression of MOG at the outer surface of the myelin sheath, accessible for autoimmune processes, is a possible explanation for its high encephalitogenicity (3, 7). Abbreviations used: N, N-terminus part of MOG; C, C-terminus part of MOG.

REFERENCES

1. Kroepfl JF, Viise LR, Charron AJ, Linington C, Gardinier MV. Investigation of myelin/oligodendrocyte glycoprotein membrane topology. Journal of neurochemistry. 1996;67(5):2219-22.
2. Johns TG, Bernard CC. The structure and function of myelin oligodendrocyte glycoprotein. Journal of neurochemistry. 1999;72(1):1-9.
3. Lee DH, Linker RA. The role of myelin oligodendrocyte glycoprotein in autoimmune demyelination: a target for multiple sclerosis therapy? Expert opinion on therapeutic targets. 2012;16(5):451-62.
4. Jilek S, Schluep M, Pantaleo G, Du Pasquier RA. MOBP-specific cellular immune responses are weaker than MOG-specific cellular immune responses in patients with multiple sclerosis and healthy subjects. Neurological sciences : official journal of the Italian Neurological Society and of the Italian Society of Clinical Neurophysiology. 2013;34(4):539-43.

5. Lindert RB, Haase CG, Brehm U, Linington C, Wekerle H, Hohlfeld R. Multiple sclerosis: B- and T-cell responses to the extracellular domain of the myelin oligodendrocyte glycoprotein. Brain : a journal of neurology. 1999;122 (Pt 11):2089-100.
6. Kim SM, Woodhall MR, Kim JS, Kim SJ, Park KS, Vincent A, et al. Antibodies to MOG in adults with inflammatory demyelinating disease of the CNS. Neurology(R) neuroimmunology & neuroinflammation. 2015;2(6):e163.
7. Brunner C, Lassmann H, Waehneldt TV, Matthieu JM, Linington C. Differential ultrastructural localization of myelin basic protein, myelin/oligodendroglial glycoprotein, and 2',3'-cyclic nucleotide 3'-phosphodiesterase in the CNS of adult rats. Journal of neurochemistry. 1989;52(1):296-304.
