# Supplementary material for: Clinical and immunological control of experimental autoimmune encephalomyelitis by tolerogenic dendritic cells loaded with MOG-encoding mRNA
Source: J Neuroinflammation. 2019 Aug 15;16:167. doi: 10.1186/s12974-019-1541-1 (PMC6696692; doi:10.1186/s12974-019-1541-1)
Supplement: Supplementary file 3 — Figure S3. Nucleotide sequence of the Sig-full length MOG-LAMP-3 and Sig-extracellular MOG-LAMP-3 constructs. (PDF 385 kb) [file 12974_2019_1541_MOESM3_ESM.pdf]

SUPPLEMENTARY FIGURE 3

A. *Sig-full length MOG-LAMP-3* vector

ACTAGTGCCACCATGGCTGCCCCTGGCGCCAGAAGGCCTCTGCTGCTCCTGCTGGCTGGCCTGGCTCATG
GCGCCAGCGCCCTGTTCGAGGACCCT**GCCTGCCTGTGGTCCTTCAGCTGGCCCAGCTGCTTCCTGAGCCTC
CTGCTCCTGCTCCTCCTGCAGCTGAGCTGCTCCTACGCCGGCCAGTTCAGAGTGATCGGCCCTGGCTACC
CCATCAGAGCCCTCGTGGGAGATGAGGCCGAGCTGCCCTGCAGAATCAGCCCCGGCAAGAACGCCACC
GGCATGGAAGTGGGCTGGTACAGATCCCCCTTCAGCCGGGTGGTGCACCTGTACAGAAACGGCAAGG
ACCAGGACGCCGAGCAGGCCCCCGAGTACAGAGGCAGAACCGAGCTGCTGAAAGAGACAATCAGCGA
GGGCAAAGTGACCCTGAGAATCCAGAACGTGCGGTTCAGCGACGAGGGCGGCTACACCTGTTTCTTCC
GGGACCACAGCTACCAAGAAGAGGCCGCCATGGAACTGAAGGTCGAGGACCCCTTCTACTGGGTCAAC
CCCGGCGTGCTGACCCTGATCGCCCTGGTGCCCACAATCCTGCTCCAGGTGTCCGTGGGCCTGGTGTTC
CTGTTCCTGCAGCACAGACTGAGAGGCAAGCTGAGAGCCGAGGTGGAAAACCTGCACAGAACCTTCG
ACCCCCACTTCCTGAGAGTGCCCTGCTGGAAGATCACCCTGTTCGTGATCGTGCCCGTGCTGGGCCCCCT
GGTGGCCCTGATCATCTGCTACAACTGGCTGCACAGAAGGCTGGCCGGACAGTTCCTGGAAGAACTGA
GAAACCCCTTC***CTGTCCGACTACACCGTGGTGCTGCCCATGGTGGCCATCATCGTGGTGGTCATCTGCGTCG
TGGGCCTGAGCGTGTACAAGATCAGACAGAGACACCAGAGCAGCGCCTACCAGAGAATC*TGA

B. *Sig-extracellular MOG-LAMP-3* vector

ACTAGTGCCACCATGGCTGCTCCAGGTGCTAGAAGGCCTCTGCTGCTTCTGCTTCTGGCTGGACTTGCTCATG
GCGCCTCTGCTCTGTTTGAGGACCCT**GGCCAGTTTAGAGTGATCGGCCCTGGATACCCTATCAGAGCCCTC
GTGGGAGATGAGGCCGAGCTGCCTTGTAGAATCTCTCCTGGCAAGAACGCCACCGGCATGGAAGTCGG
ATGGTACAGATCCCCATTCAGCAGAGTGGTGCACCTGTACAGAAACGGCAAGGACCAGGATGCTGAGC
AGGCTCCTGAGTACAGAGGCAGAACCGAGCTGCTGAAAGAGACAATCAGCGAGGGCAAAGTGACCCT
GAGAATCCAGAACGTGCGGTTCAGCGACGAAGGCGGCTACACATGTTTCTTCAGGGACCACAGCTACC
AAGAGGAAGCCGCCATGGAACTGAAGGTCGAGGACCCCTTTTACTGGGTCAACCCTGGCGTGCTGACA**
*CTGAGCGATTACACCGTGGTGCTGCCTATGGTGGCCATCATCGTGGTGGTCATCTGTGTCGTGGGCCTGAGC
GTGTACAAGATCAGACAGAGACACCAGAGCAGCGCCTACCAGAGAATC*TGA

Supplementary Figure 3. Nucleotide sequence of the *Sig-full length MOG-LAMP*-3 (**A**) and *Sig-extracellular MOG-LAMP*-3 (**B**) constructs, both codon-optimized for mus musculus. MOG nucleotide sequence was based on NCBI gene 17441. Legend: *Sig* sequence; ***MOG* sequence**; *LAMP-3* sequence.
