# Supplementary material for: Clinical and immunological control of experimental autoimmune encephalomyelitis by tolerogenic dendritic cells loaded with MOG-encoding mRNA
Source: J Neuroinflammation. 2019 Aug 15;16:167. doi: 10.1186/s12974-019-1541-1 (PMC6696692; doi:10.1186/s12974-019-1541-1)
Supplement: Supplementary file 4 — Figure S4. Graphical overview of the experimental set-up of the in vivo experiment. (PDF 279 kb) [file 12974_2019_1541_MOESM4_ESM.pdf]

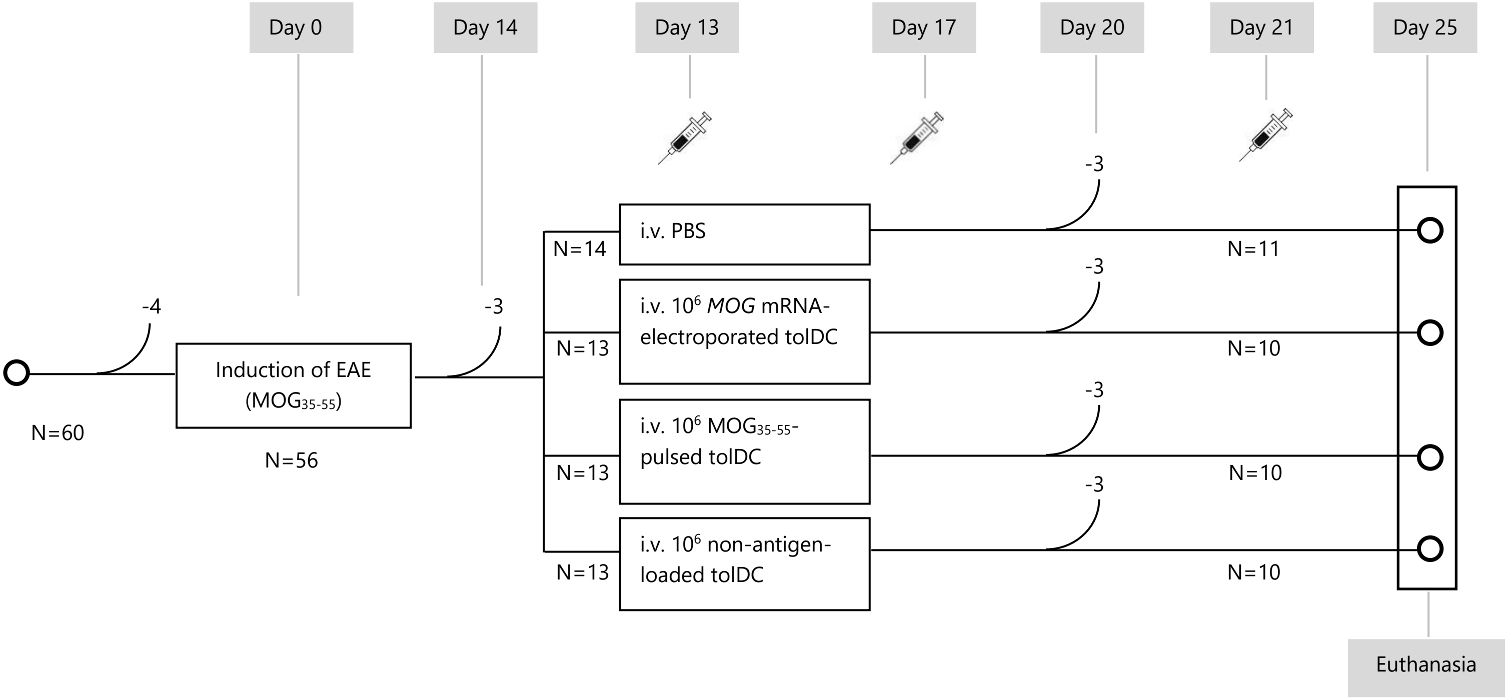

<u>Supplementary Figure 4</u>. Graphical overview of the experimental set-up of the *in vivo* experiment. Therapeutic administrations were performed at day 13 pi, day 17 pi and day 21 pi, represented by a syringe on this figure. Abbreviations used: pi, post induction; i.v., intravenously.
