# Supplementary material for: Clinical and immunological control of experimental autoimmune encephalomyelitis by tolerogenic dendritic cells loaded with MOG-encoding mRNA
Source: J Neuroinflammation. 2019 Aug 15;16:167. doi: 10.1186/s12974-019-1541-1 (PMC6696692; doi:10.1186/s12974-019-1541-1)
Supplement: Supplementary file 5 — Figure S5. Representation of individual clinical scores. (PDF 349 kb) [file 12974_2019_1541_MOESM5_ESM.pdf]

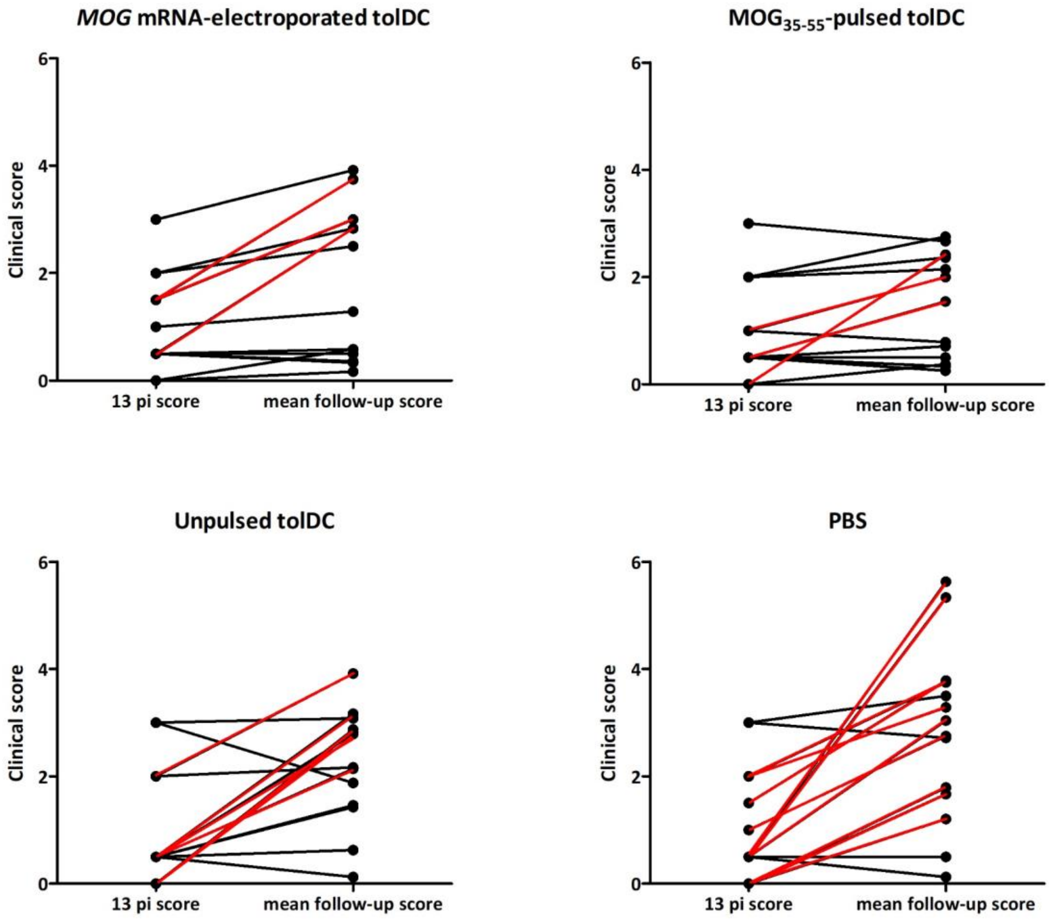

Supplementary Figure 5. Representation of individual clinical score of the initial day of treatment (day 13 pi) and the corresponding mean clinical score during the treatment period (from day 14 pi to sacrifice) from mice treated with non-antigen-loaded tolDC (n=13), MOG_{35-55}-pulsed tolDC (n=13), *MOG* mRNA-electroporated tolDC (n=13) or PBS (n=14). Non-responder mice are highlighted in red.
