# Supplementary material for: Clinical and immunological control of experimental autoimmune encephalomyelitis by tolerogenic dendritic cells loaded with MOG-encoding mRNA
Source: J Neuroinflammation. 2019 Aug 15;16:167. doi: 10.1186/s12974-019-1541-1 (PMC6696692; doi:10.1186/s12974-019-1541-1)
Supplement: Supplementary file 6 — Figure S6. Flow cytometric evaluation of the proportion of Treg, Breg and NK cells in splenocytes. (PDF 289 kb) [file 12974_2019_1541_MOESM6_ESM.pdf]

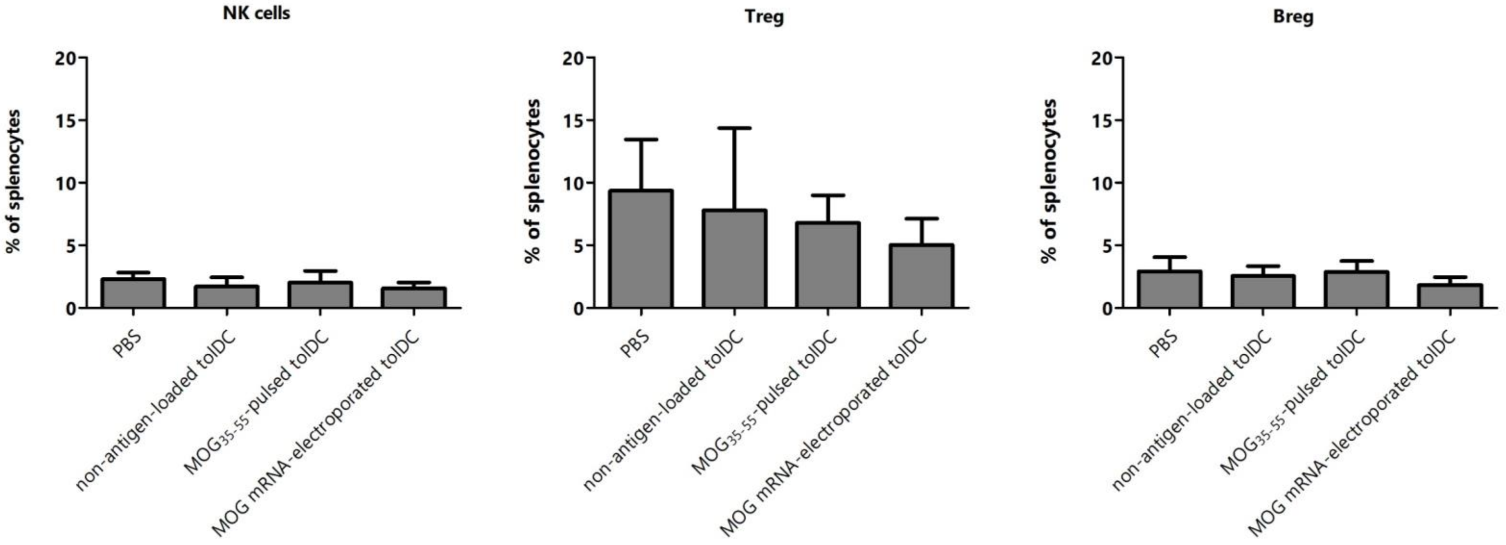

Supplementary Figure 6. Flow cytometric evaluation of the proportion of Treg (CD3+CD4+CD25+), Breg (CD19+CD5+CD1d+) and NK cells (CD3-NKp46+NK1.1+) in splenocytes of mice treated with vehicle only (PBS), non-antigen-loaded tolDC, MOG_{35-55}-pulsed tolDC or MOG mRNA-electroporated tolDC. Mice (n=9 in the PBS-treated group and n=10 in the three other treatment groups) were sacrificed at the end of the in vivo model (day 25 pi). No statistically significant difference in the proportion of regulatory immune cell subsets could be demonstrated between the different treatment groups using Kruskal-Wallis test with Dunn's post-hoc test.
