# Supplementary material for: Clinical and immunological control of experimental autoimmune encephalomyelitis by tolerogenic dendritic cells loaded with MOG-encoding mRNA
Source: J Neuroinflammation. 2019 Aug 15;16:167. doi: 10.1186/s12974-019-1541-1 (PMC6696692; doi:10.1186/s12974-019-1541-1)
Supplement: Supplementary file 7 — Table S1. Amino acid sequences of the myelin peptides used for screening for inter- and intramolecular epitope spreading. (PDF 317 kb) [file 12974_2019_1541_MOESM7_ESM.pdf]

SUPPLEMENTARY TABLE 1

Peptide	Amino acid sequence
**MBP_{4-14}**	QKRPSQRSKYL
**MBP_{84-97}**	VVHFFKNIVTPRTP
**$PLP_{139-151}$**	HCLGKWLGHPDKF
**$PLP_{178-191}$**	NTWTTCQSIAFPSK
**PLP_{56-70}**	DYEYLINVIHAFQYV
**MOG_{35-55}**	MEVGWYRSPFSRVVHLYRNGK
**MOG_{92-106}**	DEGGYTCFFRDHSYQ

Supplementary Table 1. Amino acid sequences of the myelin peptides used for screening for inter- and intramolecular epitope spreading.
