# Supplementary material for: Clinical and immunological control of experimental autoimmune encephalomyelitis by tolerogenic dendritic cells loaded with MOG-encoding mRNA
Source: J Neuroinflammation. 2019 Aug 15;16:167. doi: 10.1186/s12974-019-1541-1 (PMC6696692; doi:10.1186/s12974-019-1541-1)
Supplement: Supplementary file 8 — Table S2. Clinical data of MOG35–55 EAE mice. (PDF 393 kb) [file 12974_2019_1541_MOESM8_ESM.pdf]

SUPPLEMENTARY TABLE 2

	**PBS**	**Non-antigen-loaded tolDC**	***MOG* mRNA-electroporated tolDC**	**MOG_{35-55}-pulsed tolDC**
**Maximum score**	3.46 ± 1.59	2.81 ± 1.15	2.15 ± 1.53	2.00 ± 1.24
**Cumulative score**	32.00 ± 19.6	25.31 ± 14.2	20.38 ± 17.5	16.77 ± 11.9
**N° of responders (%)**	4/14 (28.6%)	7/13 (53.8%)	10/13 (76.9%)	10/13 (76.9%)

Supplementary Table 2. Clinical data of MOG_{35-55} EAE mice treated with PBS, non-antigen-loaded tolDC, *MOG* mRNA-electroporated tolDC or MOG_{35-55}-pulsed tolDC. Maximum score: group mean of the maximum score for each mouse. Cumulative score: group mean of the sum of daily scores for each mouse from day 7 pi onwards. Data are expressed as mean ± standard deviation. Responders: mice showing a <1-point increase in the mean clinical score compared to their respective initial clinical score at first treatment administration (day +13 pi). The mean clinical score during the treatment for each mouse was calculated as the sum of daily scores from day 14 pi to 25 pi divided by 12 (number of follow-up days after treatment). Abbreviations used: pi, post induction.
